# *Entamoeba* lysyl-tRNA Synthetase Contains a Cytokine-Like Domain with Chemokine Activity towards Human Endothelial Cells

**DOI:** 10.1371/journal.pntd.0001398

**Published:** 2011-11-29

**Authors:** Manuel Castro de Moura, Francesc Miro, Jung Min Han, Sunghoon Kim, Antonio Celada, Lluís Ribas de Pouplana

**Affiliations:** 1 Institute for Research in Biomedicine, Barcelona, Spain; 2 Center for Medicinal Protein Network and Systems Biology, College of Pharmacy, Seoul National University, Seoul, Korea; 3 Department of Immunology and Physiology, School of Biology, University of Barcelona, Barcelona, Spain; 4 Catalan Institution for Research and Advanced Studies, Barcelona, Spain; Jawaharlal Nehru University, India

## Abstract

Immunological pressure encountered by protozoan parasites drives the selection of strategies to modulate or avoid the immune responses of their hosts. Here we show that the parasite *Entamoeba histolytica* has evolved a chemokine that mimics the sequence, structure, and function of the human cytokine HsEMAPII (*Homo sapiens* endothelial monocyte activating polypeptide II). This *Entamoeba* EMAPII-like polypeptide (EELP) is translated as a domain attached to two different aminoacyl-tRNA synthetases (aaRS) that are overexpressed when parasites are exposed to inflammatory signals. EELP is dispensable for the tRNA aminoacylation activity of the enzymes that harbor it, and it is cleaved from them by *Entamoeba* proteases to generate a standalone cytokine. Isolated EELP acts as a chemoattractant for human cells, but its cell specificity is different from that of HsEMAPII. We show that cell specificity differences between HsEMAPII and EELP can be swapped by site directed mutagenesis of only two residues in the cytokines' signal sequence. Thus, *Entamoeba* has evolved a functional mimic of an aaRS-associated human cytokine with modified cell specificity.

## Author Summary

Amebiasis caused by the parasite *Entamoeba histolytica* is one of the leading causes of dysentery worldwide, with an estimated annual mortality of 100.000. In 10% of patients, the intestinal infection can spread to internal organs causing hepatic, lung, and brain abscesses. Little is known about the strategies used by the parasite to evade or minimize the inflammatory and immune responses of its host. In this manuscript we report the discovery that *Entamoeba* has evolved a polypeptide that functionally mimics the activity of a human cytokine (EMAPII) involved in the regulation of inflammation. This polypeptide termed EELP (*Entamoeba* EMAPII-Like Polypeptide) is capable of attracting human cells, just like its human counterpart but, unlike EMAPII, EELP does not act on inflammatory cells. We have characterized the dynamics of gene expression that regulate EELP synthesis, and we demonstrate that the protein is produced when *Entamoeba* encounter inflammation signals produced by their human host. Our working hypothesis is that EELP is used by the parasite to shield itself from human inflammation. In general, the discovery of EELP opens a new avenue of research into the mechanisms used by *Entamoeba* to survive their host's environment.

## Introduction

*Entamoeba histolytica* is an amitochondriate unicellular protozoan and the leading cause of dysenteric human deaths in the world [1]. The infection cycle of *E. histolytica* involves the colonization of the gut and, in more severe cases, the penetration of the gut ephitelium by the parasite. Crossing of the epithelial barrier in the gut relies on the ability of the pathogen to induce local inflammation and apoptosis of epithelial cells [2], [3], [4]. Once the parasite has traversed the epithelial gut barrier it can reach internal organs and cause systemic infections and internal lesions such as amebic liver abscesses (ALA) [5].

ALA formation starts with the interaction of *Entamoeba* trophozoites with liver endothelial cells [6] that triggers cytokine production and the recruitment of neutrophils and macrophages around each individual parasite, forming a granuloma [7], [8]. This immune response causes acute inflammation and massive trophozoite killing [9], while surviving amoebae divide causing abscesses [10]. In general, host immune responses during protozoan infections are met by passive and active strategies evolved by the parasites to modulate and minimize their effect [4], [11], [12]. For example, *Entamoeba* MLIF (Monocyte Locomotion Inhibitory Factor) is an immunosupressor pentapeptide that is released by the ameba to disturb cytokine and chemokine production by host immune cells [13].

Aminoacyl-tRNA synthetases (aaRS) are multi-domain proteins responsible for the aminoacylation of transfer RNAs. In many species aaRS, and homologous proteins, are implicated in other metabolic pathways, cell signaling mechanisms, and developmental processes [14], [15], [16]. These non canonical aaRS functions are often carried out by newly evolved domains, particularly in mammalian enzymes [17]. For example, WHEP domains in mammalian Glutamyl-Prolyl-tRNA synthetase control gene expression after phosphorylation triggered by interferon-γ (IFN-γ) [18], [19], [20]. These new activities may require the proteolytic processing of the aaRS, or an alternative splicing of their genes [21], [22], [23]. Non canonical aaRS activities have also been identified in protozoans [24].

Eukaryotic cytoplasmic aaRS form a multi-enzyme complex composed of up to nine individual aaRS [25]. aaRS complexes are structurally stable and assemble around three additional proteins, known as aaRS complex-inteacting multifunctional proteins 1–3 (AIMP1, AIMP2, and AIMP3), which also act as cytokines [26], [27], [28]. AIMP1 can be proteolytically cleaved from the complex to generate a protein known as endothelial monocyte activating polypeptide II (EMAPII) [29]. EMAPII was first characterized as a secreted cytokine from mouse tumors and has since been reported to be active in a large number of cell signaling and developmental pathways [30]. In humans, EMAPII (HsEMAPII) is a wide acting cytokine that induces apoptosis and migration in endothelial cells, and migration of inflammation related cell types, like macrophages and monocytes.

Homologous sequences to EMAPII can be found as domains of mammalian tyrosyl-tRNA synthetases (YRS). EMAPII-like domains in mammalian YRS are proteolytically cleaved and act as cytokines (HsCtYRS) [23]. EMAPII-like domains are also found in bacterial methionyl-tRNA synthetases, where they play a role in tRNA^Met^ aminoacylation [31].

We have discovered that, in the genus *Entamoeba*, methionyl- and lysyl-tRNA synthetases (EhMRS and EhKRS) share an almost identical C-terminal domain (99% sequence identity in their last 166 amino acids). We have labeled these domains as EhCtMRS (residues 588 to 754 of EhMRS) and EhCtKRS (residues 603 to 769 of EhKRS), respectively. Due to their extreme sequence identity, we will generally refer to these domains as *Entamoeba* EMAPII-like polypeptide (EELP). This domain is also highly similar to HsEMAPII. To our knowledge, this is the first example of two aaRS of different classes [32] containing a structurally identical domain, and the first report of an EMAPII-like domain attached to a KRS.

The cytokine activities of aaRS-related domains have been partially characterized in mammals, but no cell signaling activity has been reported for this kind of molecule in unicellular eukaryotes. Moreover, the actual biological role of EMAPII remains controversial. Here we characterize the role of the EMAPII-like domains found in *Entamoeba*. Our results show that the two copies of EELP are closely co-evolving in a process unrelated to the tRNA aminoacylation reaction. These domains are localized to the surface of the parasite, and the full length aaRSs that initially contain them are proteolytically processed to produce the isolated EELP domains.

We demonstrate that EELP displays a chemokine activity for human cells at concentrations comparable to those required for HsEMAPII. The cellular tropism of EELP is different from that of HsEMAPII, because EELP readily attracts human endothelial cells but not monocytes. This difference can be reversed by the mutation of two single residues in the signaling motif of these chemokines. We propose that EELP is a functional mimic of HsEMAPII evolved to modulate the cellular environment encountered by the parasite during infection with the purpose of escaping the immune response of the host.

## Methods

### Reagents and plasmids

*Entamoeba histolytica* genomic DNA was a kind gift of Dr. Mario A. Rodriguez (Department of Genetics and Molecular Biology, Cinvestav-IPN). L-[^3^H] lysine, and HisTrap nickel columns were from Amersham Biosciences. Restriction enzymes were from New England Biolabs. Expression vector pET-30 Ek/LIC was from Novagen. Pfu Ultra DNA polymerase and XL-10 Gold cells were from Stratagene. pET-20b(+)/C-TyrRS coding for the C-terminal domain of human tyrosyl-tRNA synthetase was a kind gift from Dr Schimmel's lab. *Drosophila melanogaster* muscle protein 20 (DmMp20) was purchased from Drosophila Genomics Resource Center and cloned into pET-30 Ek/LIC. Recombinant human EMAPII (rhEMAPII), tumor necrosis factor-α (TNF-α and vascular endothelial growth factor (VEGF) were from Peprotech and lipopolysaccharide (LPS) from Sigma. Affinity purified antibodies α-EhNtKRS (raised against the peptide sequence 91-YENKEDFVSLTKMIYRGDIC-110) and α-EELP (raised against the peptide sequence 741-CLVRTNDVPLIVKDTEL-756 of EhKRS) were provided by AntibodyBcn. The α-EELP recognizes EELP domain present in both EhKRS and EhMRS..α-LGL antibody was a kind gift from Dr. David Mirelman (Department of Biological Chemistry, Weizmann Institute).

### Cell lines

*Entamoeba histolytica* trophozoites (HM-1:IMSS) were grown axenically in LYI-S-2 (Trypticase-yeast extract iron serum) medium supplemented with 100 U of penicillin/ml and 100 µg of streptomycin sulfate/ml at 37°C [33]. Trophozoites were harvested during log-phase growth by incubation on ice for 5 minutes, centrifugation at 250×g for 5 minutes and washed in PBS three times. HUVEC were purchased from Lonza and used till passage 6 with EGM2 medium (Lonza). Monocytes were purified from blood of healthy volunteers by Ficoll-paque centrifugation gradient after leaving to adhere to plastic culture flasks for 2 hours in RPMI 1640 medium (Invitrogen) supplemented with heat-inactivated 10% fetal bovine serum (FBS). DLD1 cells were maintained in DMEM medium supplemented with decomplemented 10% FBS. Total human blood was provided by the Barcelona blood bank (Banc de Sang i Teixits, www.bancsang.net) after approval of the project by the local ethic committee (PR(BST)22/2010).

### Quantitative RT-PCR

In a six well plate, 1.5×10^6^ amoebas were stimulated with 100 ng/ml TNF-α or 100 ng/ml LPS for 6 hours. In other series of experiments 1.5×10^6^ trophozoites were co-cultured with the same number of DLD1, HUVEC or monocytes, activated or not with 100 ng/ml LPS for 6 hours. Total RNA was extracted with Trizol Reagent (Invitrogen Life technologies) following the protocol described by the manufacturer. cDNA was obtained from 2 µg of RNA by using the Reverse Transcription System from Promega. Quantitative RT-PCR were done in a StepOnePlus instrument and using Power SYBR Green detection system from Applied Biosystems. Primers used are detailed in Methods S1. The relative gene expression was determined by calculating ΔC_t_ values between *EhkrS* or *EhmrS* genes and that of *EhtrS* (coding for *E. histolytica* threonyl-tRNA synthetase). The values were normalized to non stimulated trophozoites.

### *Entamoeba* lysates, Cellular fractionation and western blotting

0.25×10^6^ amoebas (per well) were stimulated for 24 hours with 100 ng/ml TNF-α, or 100 ng/ml LPS or incubated with 0.5×10^6^ monocytes or DLD1 cells previously activated or not with 100 ng/ml of LPS for 6 hours, in a six-well plate. Cells were washed in PBS pH 6.8, collected by centrifugation and resuspended in PBS containing the protease inhibitors 10 mM 2-hydroxymercuribenzoate, 100 µM E-64, 5 mM N-ethylmaleimide (NEM), 6 mM Benzamidine, 0.2 mM Leupeptin, 2 mM phenylmethylsulfonyl fluoride (PMSF), 1 mM EDTA, 1 mM EGTA and 1% Triton X-100. The trophozoites were lysed by four freeze-thaw cycles. The debris and unbroken cells were removed by centrifugation at 250× g for 5 minutes and supernatant was further spun at 100,000× g for 1 hour at 4°C. For cellular fractionation studies the cell pellet was resuspended at 10^6^/ml in the PBS buffer plus protease inhibitors but without detergent. Cells were lysed and centrifuged as described above and the membrane fraction was collected by ultracentrifugation at 100,000× g for 1 hour at 4°C. The membrane pellet was resuspended in PBS buffer plus protease inhibitors and 1% Triton X-100. For protein processing assays, protein extracts were prepared in the same way, but without adding protease inhibitors to the extract. Protein concentrations were calculated with Bradford reagent (Bio-Rad). 35 µg of total protein per sample was resuspended in Laemmli buffer, separated by 12% SDS-PAGE and immunoblotted to polyvinyldifluoride (Immobilon-P, Millipore) membranes.

### Immunoprecipitation and proteomics analysis

A total of 6×10^6^ amoebas were lysed using 1% Triton X-100 containing buffer as described above. Crude extracts were clarified by centrifugation at 10,000× g for 10 minutes and further precleared with 35 µl of protein G-Sepharose beads for 1 hour at 4°C. Two additional preclearings were performed. For immunoprecipitations, precleared lysates were incubated overnight at 4°C with 2 µg of the affinity purifed α-EELP antibody, or with 10 µl of pre-immune serum. Subsequently, 30 µl of protein G-Sepharose beads were added to the lysates for 3 additional hours. Proteins in the immunoprecipitates were separated by SDS-PAGE and coomassie blue stained. Coomassie stained bands were cut and analyzed by the Proteomics facility of the Barcelona Science Park and the Mass Spectrometry core facility of the Institute for Research in Biomedicine. Samples were trypsinised, and the resulting peptides were sequenced by nano-LC-MS/MS obtaining different fragmentation spectra. A database search was performed with Proteome Discoverer software v1.2.0.208 (Thermo) using Sequest and Mascot engines and SWISSPROT and NCBI nr databases. Peptide mass tolerance was 10 ppm and the MS/MS tolerance was 0.8 Da. Only proteins containing peptides with confidence higher than 95% were considered.

### Confocal microscopy

Trophozoites on prewarmed cover slips were fixed with 4% (w/v) paraformaldehyde for 1 hour at 37°C, incubated with 0.1 M glycine in PBS for 15 minutes at room temperature, permeabilized with 0.2% (v/v) Triton X-100 for 15 minutes and blocked with 1% (w/v) BSA in PBS at 37°C for 30 minutes. Samples were incubated with chicken α-NtEhKRS (1∶1000) and rabbit α-EELP (1∶500) antibodies for 1 hour. Subsequently, cells were washed with PBS and incubated with alexa fluor 555-labeled goat α-rabbit and alexa fluor 488-labeleld goat α-chicken secondary antibodies (Molecular Probe; 1∶1000) in blocking solution at room temperature for 1 hour. Finally, cells were washed and counterstained with DAPI 1 µg/ml (Sigma), and pictures were taken with a Spectral Confocal Microscope Leica SP5. Fluorescence intensity was quantified with ImageJ free software.

### DNA amplification and cloning

Genomic DNA from different *Entamoeba* species was obtained using Puregene Tissue Core Kit B (Qiagen). Amplification of *krS* and *mrS* genes from *Entamoeba nuttallii* (accession number HQ121505, HQ121508), *Entamoeba moshkovskii* (HQ121506, HQ121507) and *Entamoeba terrapinae* (HQ121509) was performed using primers (see Methods S1) designed from the alignment of known *Entamoeba krS* and *mrS* genes. The 2307 base pair gene coding for *E. histolytica KRS* (GenBank XM645508) was amplified by PCR from *E. histolytica* genomic DNA using Pfu Ultra DNA polymerase (see Methods S1 for primer sequences). The amplified DNA fragment was cloned into the pET-30 Ek/LIC vector to yield plasmid pET-30-EhKRS. The Ct region of the gene and the gene without the EMAPII-like domain were also amplified and cloned into the pET-30 Ek/LIC vector to yield pET-30-EhCtKRS (also named pET-30-EELP) and pET-30-EhKRSΔCt plasmids, respectively. The correct sequence and orientation of the genes were checked by sequencing the constructions entirely. Mutated constructs were performed with QuickChange Site-directed mutagenesis kit (Stratagene) following the manufacturer's protocol.

### Enzyme overexpression and purification

*Escherichia coli* BL21 (DE3) cells transformed with the pET-30 constructs were grown at 37°C to an optical density A_600 nm_ = 0.6. Protein expression was induced with 1 mM IPTG for 4 hours at 37°C. Initial purification on nickel affinity columns was performed using standard procedures at 4°C. Binding and wash buffer had a room temperature pH of 7.8 and contained 50 mM Tris/HCl, 100 mM NaCl buffer, and 20 mM imidazole. Elution buffer was the same but it contained 500 mM imidazole. Subsequent visualization on coomassie blue stained 12% polyacrylamide gel showed the protein preparations were over 99% pure. The protein solutions were dialyzed in pyrogen-free buffer (50 mM TrisHCl, 100 mM NaCl, 50% Glycerol), passed through polymyxin resin (Pierce) and treated with Triton X-114 (Calbiochem) at final concentration of 1% [34] to eliminate endotoxins. Finally, proteins were passed through a 0.22 µm PES filter. Protein concentrations were determined by Bradford assay [35]. For the aminoacylation assays, final concentrations of the EhKRS and EhKRSΔCt proteins were determined by active site titration as described in the literature [36].

### Protein processing assays

Purified EhKRS was digested with different concentrations of *E.histolytica* extracts or human leukocyte elastase (Sigma) for 5, 15 or 30 minutes at 37°C in the presence or absence of protease inhibitors. After treatment samples were mixed with Laemmli buffer and heated at 90°C for 5 minutes to stop the reaction. Samples were loaded onto a 12% SDS-PAGE gel and analyzed by immunobloting. Edman degradation analysis of these samples was performed at the Proteomics and Bioinformatics facility of Universitat Autònoma de Barcelona, a member of ProteoRed network, to identify the point or points of proteolytic cleavage of the purified protein.

### tRNA substrate preparation

Constructions containing a T7 promoter fused with the tRNA genes of interest were obtained and *in vitro* transcribed using T7 RNA polymerase and according to standard protocols [37]. Transcripts were separated on denaturing PAGE, full-length tRNAs were eluted from the gel using an electroelution apparatus (Schleicher & Schüll) and refolded (80°C followed by gradual cooling in the presence of 1 mM MgCl_2_). Finally, aminoacylation plateaus were used to calculate the concentration of active molecules for each tRNA preparation.

### Aminoacylation assays

Aminoacylation of tRNA was performed at 37°C in 100 mM Hepes, pH 7.2, 6 µM lysine, 30 mM MgCl_2_, 30 mM KCl, 0.5 mM DTT, 5 mM ATP, 0.1 mg/ml BSA, 500 Ci/mol L-[^3^H] lysine and varying concentrations of tRNA transcripts (0.5–20 µM). Reactions were initiated by addition of pure enzyme and samples of 22 µl were spotted onto Whatman 3MM discs at varying time intervals. Radioactivity was measured by liquid scintillation. Enzyme concentrations were experimentally determined in order to obtain linear velocities for kinetic constant determination. Kinetic constants were obtained from Lineweaver-Burk plots using a minimum of two independent measurements and five tRNA concentrations.

### Computational analyses

Initial database searches were performed with BLAST [38] in the NCBI public databases (http://www.ncbi.nlm.nih.gov/). Additional searches were performed at the *Entamoeba* genome project [39] websites (www.tigr.org and www.sanger.ac.uk). Protein sequence alignments were performed with CLUSTALW [40]. Phylogenetic trees of EELP amino acids sequences were calculated using the packages PHYLIP [41], PHYML [42], [43] and MrBAYES [44]. Maximum parsimony and distance trees were calculated with the programs PROTPARS and PROTDIST, and their numerical robustness was measured by bootstrap with the program CONSENSE. Maximum likelihood trees were calculated with the program PHYML and evaluated by bootstrap analysis. Bayesian phylogeny was inferred with MrBAYES.

### Cell migration assays

Cells were resuspended in chemotaxis media (RPMI 1640 containing 0.5% FBS for monocytes and EBM-2 basal medium for HUVEC) at a concentration of 1×10^6^ cells/ml. Recombinant human EMAPII (rhEMAPII), HsCtYRS, EhKRS, EhKRSΔCt, and EELP were diluted in chemotaxis medium and placed in the lower well of a 24-well transwell Boyden chemotaxis plate (Costar). The concentration of the stimuli was tipically 1 nM. In some experiments of monocyte migration, supernatants from HUVEC cells cultured with 1 nM EhKRS, EhKRSΔCt, EELP or EhMRS proteins were placed in the lower well of a chemotaxis plate. 200 µl of cells (0.2×10^6^ cells/ml) were plated in the upper well. The polycarbonate membranes separating cells from lower wells had 5 µm pore diameter for monocytes and 8 µm for HUVEC. The filled chemotaxis transwell plates were incubated at 37°C in a humidified CO_2_ incubator for 3 hours for monocytes and 16 hours for HUVEC cells. After incubation, non migratory cells on the upper surface of the filter were removed by wiping with a cotton swab, and migratory cells were fixed and stained with hemacolor (Merck). The number of migrated cells was counted by taking photographs under inverted light microscope using a 20× objective and migration was plotted either as mean of number of cells in each high power field (HPF) or as migration index (MI = number of migrating cells in each condition/number of migrating cells in basal medium). Data are presented as Mean ± SEM from at least three independent experiments. The data significance was analyzed by mean of t tests.

### Calcium flux assays

HUVEC and monocytes were stained with Fluo-4 by using the Fluo-4 NW calcium assay kit from Molecular Probes and following the manufacturer's protocol. Cells were stimulated during data acquisition with 10 nM rhEMAPII, HsCtYRS, EhKRS, EhKRSΔCt, EELP or HumEELP. For monocytes, intracellular calcium flux was recorded in a XL cytometer (Coulter) for 240 seconds and expressed as relative fluorescence units (RFU) versus time acquisition. Data were analyzed with FlowJo software (Tree Star, Ashland, OR). For HUVEC, calcium influx was monitored in a Spinning Disk microscope (Perkin Elmer) and data analyzed with Volocity software (Perkin Elmer).

### Cell Binding Assay

Monocytes and HUVEC cells (0.3×10^6^/well) were seeded onto six-well dishes. 100 nM EELP, HumEELP, HsCtYRS or DmMp20 (*Drosophila melanogaster* muscle protein of 25 kDa; see Figure S1A and C) were added to the culture medium for 60 minutes at 37°C. After that, the cells were harvested, washed three times with cold PBS, and lysed in lysis buffer (25 mM Tris/HCl, pH 7.4; 150 mM NaCl; 5 mM EDTA; 1 mM DTT; 0.5% Triton X-100; Complete proteases inhibitors (Roche Molecular Biochemicals)). The extracted proteins (35 µg) were resolved by SDS-PAGE, transferred to Immobilon membranes and blotted with anti-His antibody (Amersham Biosciences).

## Results

### Genomic analysis of *Entamoeba* species

**Figure 1:**
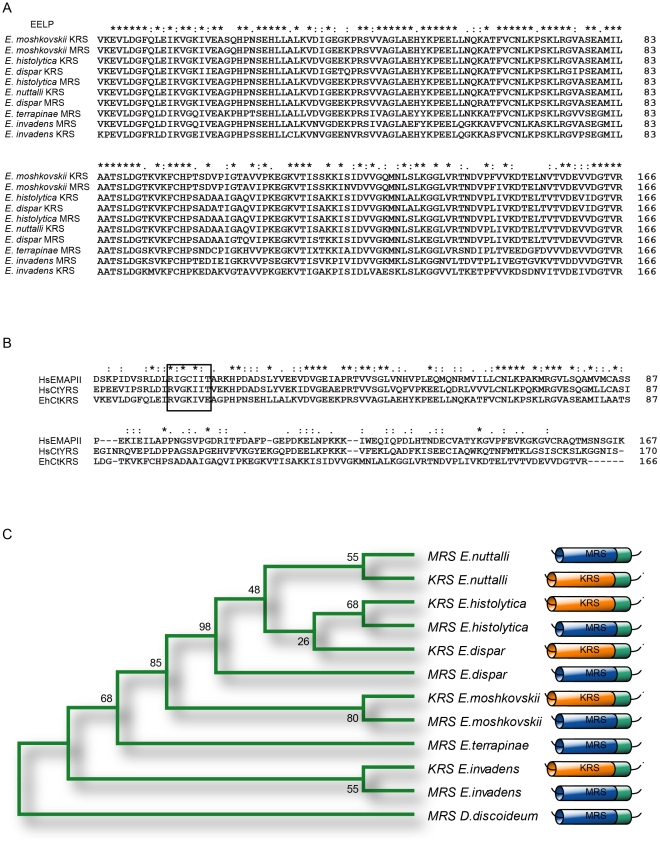
Bioinformatic analysis of EELP. (A) Protein alignment of EELP sequences from different *Entamoeba* species. (B) Protein alignment of mature EMAPII from *Homo sapiens* (HsEMAPII), C-terminal EMAPII-like domain of human tyrosyl-tRNA synthetase (HsCtYRS) and C-terminal domain of *Entamoeba* lysyl-tRNA synthetase (EhCtKRS). The boxed sequence corresponds to the heptapeptide migration motif. (C) Phylogenetic analyses of EELP protein sequences from different *Entamoeba* species. Numbers on each branch correspond to bootstrap values in the distance consensus tree.

The analysis of available genomic data for the *Entamoeba* species *E. histolytica*, *E. dispar* and *E. invadens*, and of new sequences obtained by us through the sequencing of *E. nuttalli, E. moshkovskii and E. terrapinae* genomic DNA (**Figure 1A** **and Figure S1B**) clearly shows that *Entamoeba* species contain a DNA sequence coding for a HsEMAPII-like domain in the KRS and MRS genes (**Figure 1B**). In all the *Entamoeba* species analyzed these domains are located at the C-terminal of KRS and MRS. In the case of *E. histolytica* the two domains are 99% identical to each other.

The C-terminal domain of *Entamoeba* MRS (EhCtMRS) can be found in many other species, including *Dictyostelium discoideum*, a slime mould considered one of the closest known relatives of *Entamoeba*. However, *Entamoeba* KRSs represent the only known example of a KRS containing this type of domain in their structure. The high sequence identity shared by the two domains, and the fact that the KRS domain is unique to *Entamoeba*, support the idea that EhCtKRS resulted from a duplication of EhCtMRS that integrated into the *Entamoeba* KRS gene after *Entamoeba* separated from the slime molds.

We computationally tested whether EhCtKRS and EhCtMRS are under the same evolutionary constraints, or whether they are functionally separated. All the phylogenetic trees calculated clearly show that the evolution of the domains is intra-specific and not linked to enzyme type (**Figure 1C**).

### EhCtKRS is dispensable for EhKRS aminoacylation activity

The kinetic analyses of EhKRS and the truncated variant EhKRSΔCt confirmed the limited role of the EELP in the aminoacylation reaction catalyzed by EhKRS (**Table 1**). Ablation of EELP from EhKRS (EhKRSΔCt) has a moderate effect on aminoacylation, reducing the Vmax/Km ratio approximately forty-fold. Although this reduction is significant, EhKRSΔCt maintains a level of activity well above that commonly documented to be required for cellular viability [45], [46], [47]. This fact, together with the results from our computational analyses and the almost identical sequence of the EELP domains from EhMRS and EhKRS, indicate that these two domains are likely performing additional functions beyond protein synthesis.

**Table 1:** EhCtKRS is dispensable for KRS activity.

	Vmax (s^−1^)	Km (µM)	Vmax/Km	tRNA^Lys^
EhKRS	0.18±0.09	5.23±0.007	0.034	*E. histolytica*
EhKRSΔCt	0.025±0.006	30.96±5.7	0.00081	*E. histolytica*

Kinetic parameters for EhKRS and EhKRSΔCt proteins.

### EhKRS and EhMRS, but not other aaRSs, are upregulated by inflammation signals

**Figure 2:**
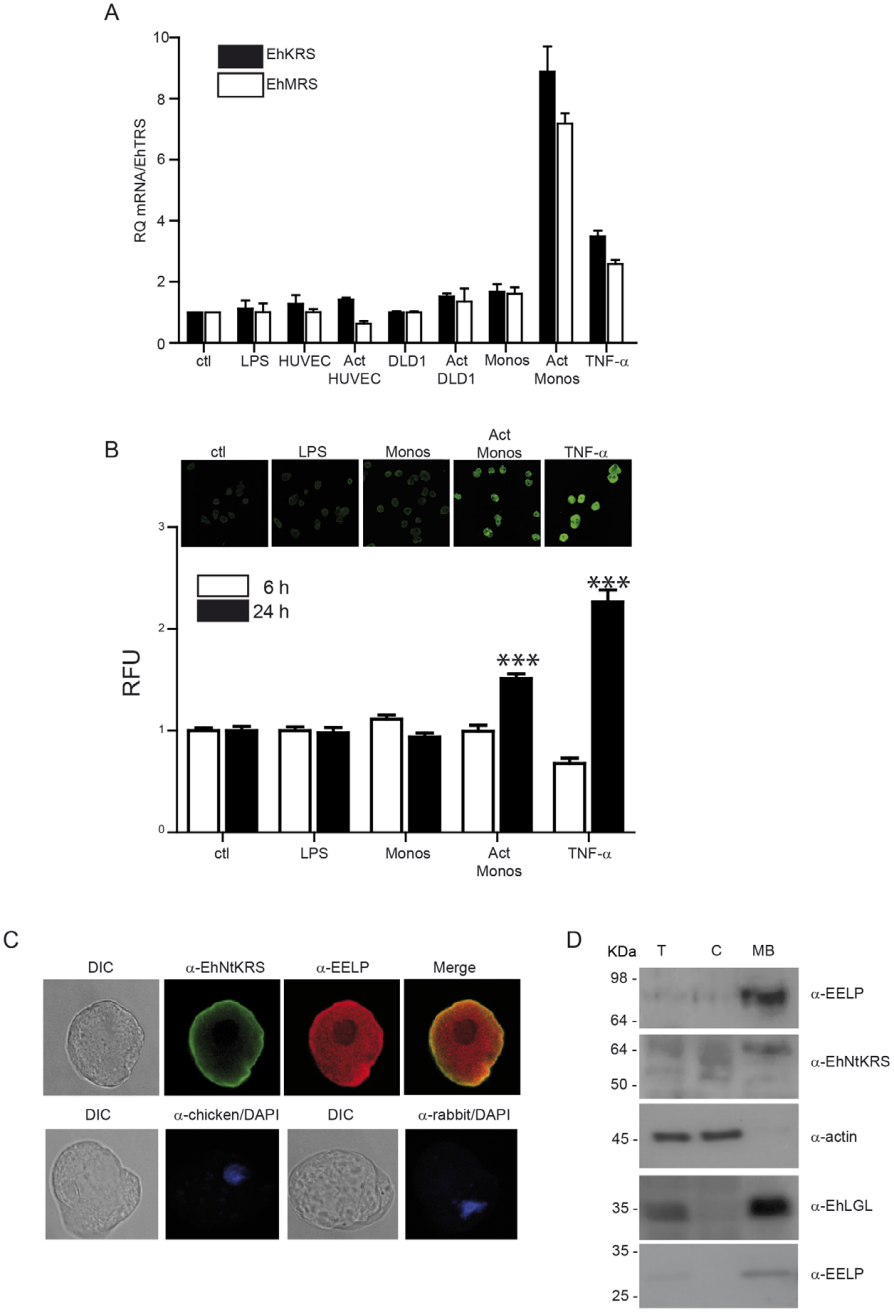
EhKRS and EhMRS are up-regulated by inflammation signals. (A) qRT-PCR of *EhKRS* (closed bars) and *EhMRS* (open bars) gene expression relative to *Entamoeba histolytica threonyl-tRNA synthetase* (*EhTRS*). Trophozoites stimulated with 100 ng/ml LPS or 100 ng/ml TNF-α or co-cultured with endothelial cells (HUVEC), colonic epithelial cells (DLD1), or primary monocytes (Monos) pre-activated (Act) or not with 100 ng/ml LPS for 6 h. Values are relative to non-stimulated amoebae (ctl) and depicted as mean ± SD from at least two independent experiments performed in triplicate. (B) Quantification of EhKRS protein expression in trophozoites stimulated for 6 hours (open bars) and 24 hours (closed bars) with 100 ng/ml LPS or 100 ng/ml TNF-α or co-cultured with monocytes (Monos) or monocytes previously activated with LPS for 6 hours (Act Monos). The fluorescence units were normalized to non-stimulated (ctl) trophozoites and depicted as mean ± SD from three different experiments. The number of cells counted per each condition was n = 40. (*** p<0.0001 vs ctl). Inserts show microphotographs of immunodetection of EhKRS protein (α-NtEhKRS) in trophozoites at 24 hours of stimulation. (C) Immunolocalization of EhKRS in *Entamoeba* trophozoites using affinity purified antibodies against EhKRS N-terminal (α-NtEhKRS) and C-terminal (α-EELP) domains. Controls of secondary antibodies merged with DAPI are shown in bottom row. (D) EhKRS cellular localization was evaluated by immunoblot analysis of *E. histolytica* lysates, marked as T (whole cell lysate), C (cytoplasmic fraction), and MB (membrane bound fraction). Specific antibodies of cytoplasmic fraction (α-actin) and membrane fraction (α-LGL) were used. α-EELP antibody recognizes a full length protein (top panel) and a C-terminal cleaved product (bottom panel). α-NtEhKRS (1∶250), α-EELP (1∶50), α-LGL (1∶1000) and α-actin (1∶1000) antibody dilutions were used.

It has been shown that the expression of human aaRS involved in cell-signaling functions responds to cytokine signals [48]. For example, human AIMP1, an aaRS-associated protein, is up-regulated by tumor necrosis factor-α (TNF-α) [49]. To test whether EhKRS or EhMRS were responsive to human inflammatory signals we analyzed the transcription levels of the genes coding for both proteins by quantitative RT-PCR in parasites treated with human TNF-α. EhKRS and EhMRS mRNAs, but not threonyl-tRNA synthetase (EhTRS) mRNA, were up-regulated roughly 3 times in cells treated with TNF-α (**Figure 2A**).

Thus, EhKRS and EhMRS seem to be regulated differently from other aaRS, and their expression is increased when the parasite is exposed to inflammatory cytokines. We also quantified EhKRS and EhMRS gene expression in co-cultures of amoebae with DLD1 colonic epithelial cells, endothelial (HUVEC) cells, or human primary monocytes. EhKRS and EhMRS were significantly and specifically up-regulated in contact with lipopolysaccharide (LPS)-activated monocytes. EhKRS up-regulation was confirmed by immunofluorescence (**Figure 2B**) and western blot (**Figure S2**) analyses. All together these data demonstrate that EhMRS and EhKRS are specifically up-regulated when amoebae are exposed to host-induced inflammatory signals.

### Molecular identification and cellular localization of EELP

Confirmation of the existence of EELP as standalone domain after cleavage from EhKRS or EhMRS was experimentally determined. Crude extracts of amoebas were immunoprecipitated with the anti-EELP antibody, the immunoprecipitated material was resolved by SDS-PAGE electrophoresis (**Figure S3A**), and proteins bands with molecular weight approximating the expected size of EELP were sequenced by mass spectrometry (**Figure S3B and C**). Indeed, a protein band approximating the molecular weight of recombinant EELP was identified whose sequence unambiguously corresponds to EELP, proving that EELP is produced by *Entamoeba* as a processed product of full length MRS and/or KRS.

We then investigated the cellular localization of EELP with antibodies against the N- and C-terminal sequences of EhKRS (α-EhNtKRS and α-EELP, respectively). Due to their high sequence similarity in the EELP region, both EhMRS and EhKRS are recognized by α-EELP. On the other hand, α-EhNtKRS specifically recognizes EhKRS. The α-EhNtKRS antibody clearly stained the *Entamoeba* surface, while the α-EELP antibody stained both the plasma membrane and the cytosol (**Figure 2C**). Immunoblot analyses of *E. histolytica* cellular fractions revealed a band of ∼90 kDa and another of 27 kDa reactive with the α-EELP antibody, and a band of ∼68 kDa reactive with the α-NtEhKRS, all of them present in the membrane fraction of the parasite's extracts (**Figure 2D**). Thus, three forms of EhKRS are found in membrane associated material: full-length EhKRS, a C-terminally cleaved EhKRS, and the cleaved C-terminal domain. This opens the possibility that the C-terminal domain of EhKRS is cleaved and released from the full-length enzyme at the surface of the parasite.

### Protease processing releases the EELP domain

**Figure 3:**
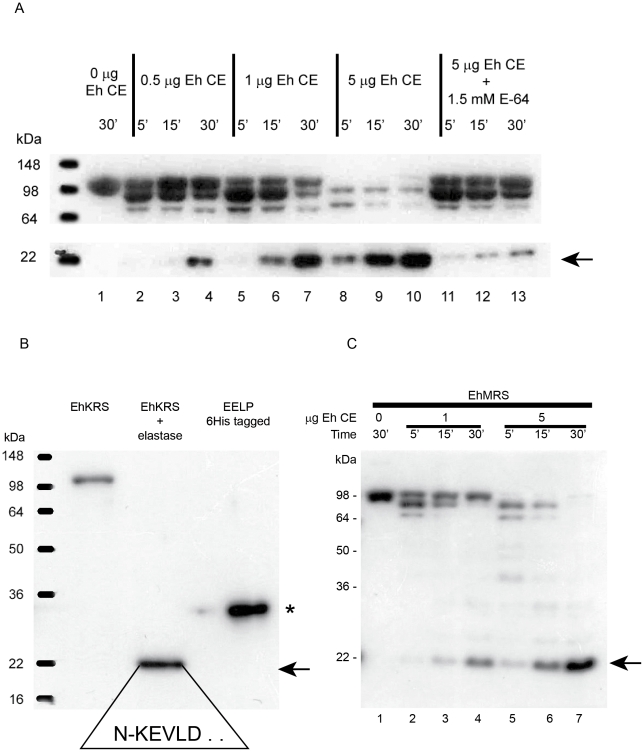
EhKRS protease processing. Immunoblots analysis with α-EELP antibody of recombinant EhKRS protein digestions using *Entamoeba* lysates (A) or human leukocyte elastase (B). (A) 1 µg recombinant EhKRS was incubated at 37°C for 30 minutes without *E. histolytica* crude extract (Eh CE; lane 1) or with 0.5 µg Eh CE (lane 2–4); 1 µg Eh CE (lane 5–7); 5 µg Eh CE (lane 8–10) or 5 µg Eh CE plus protease inhibitors (lane 11–13) for 5, 15 or 30 minutes. (B) Digestion of 1 µg recombinant EhKRS protein with elastase for 30 minutes at 37°C. (C) EhMRS protease processing. Digestion of 1 µg recombinant EhMRS at 5, 15 and 30 minutes with 1 µg of Eh CE (lane 2–4) or 5 µg Eh CE (lane 5–7). Recombinant EhMRS control without Eh CE (lane 1). Digestion products were detected by immunoblot using α-EELP antibody. Arrow shows EELP product resulting from recombinant EhKRS or EhMRS. Asterisk denotes recombinant EELP with 6 His tag plus 36 amino acids at the N-terminal. Boxed sequence corresponds to the N-terminal sequence of EELP after digestion from EhKRS by elastase, as determined by Edman degradation.

To study the possible processing of EhKRS we treated purified recombinant enzyme with a protein extract from *Entamoeba* (which contains parasite proteases) (**Figure 3A**), and with mammalian elastase (**Figure 3B**). We observed that EhKRS is cleaved readily, releasing a protein band of 22 kDa recognized by anti-EELP antibodies. Edman degradation analysis of elastase digestion products showed that first amino acids present in our sample were KEVLD (**Figure 3B**), confirming the cleavage of EhKRS and the production of EELP. We also observed that this process also applies to EhMRS (**Figure 3C**). Processing is blocked by cysteine protease inhibitors. Thus, EELP can be proteolitically cleaved from EhKRS and EhMRS by either parasite or host proteases.

### EELP mimics the chemotactic effect of human EMAPII

**Figure 4:**
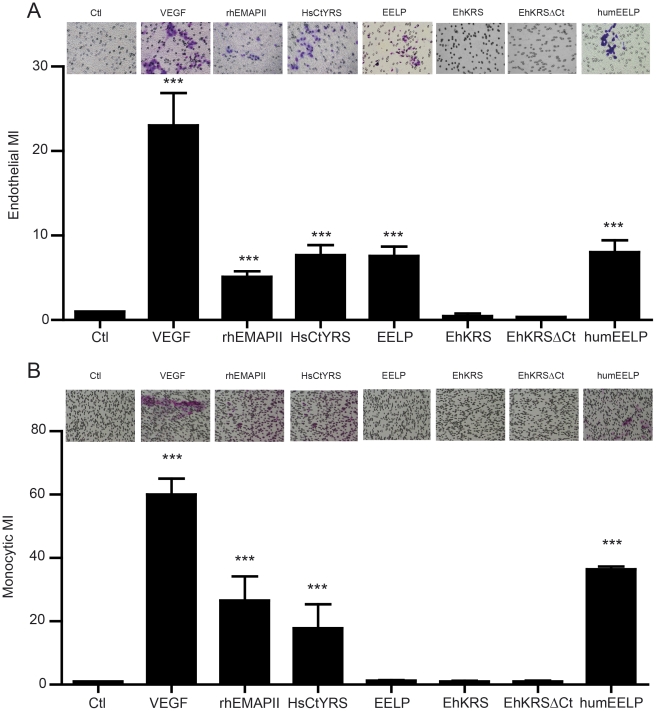
Effect of EELP on host cell migration. (A) Endothelial cell and (B) monocyte chemotaxis towards different EMAPII bearing proteins. VEGF (1 nM) was used as a common chemoattractant for both cells. Data are the mean ± SEM for at least three different experiments. Inserts show a representative photograph of cell migration. Pore membranes for HUVEC and monocyte migration were of 8 µm diameter and 5 µm diameter, respectively. Cells are stained in purple. Migration is plotted as Migration Index (MI; number of cells migrating in each condition/number of cells migrating in basal medium). Ctl, basal medium in the lower well; VEGF, vascular endothelial grothw factor; rhEMAPII, recombinant human EMAPII; HsCtYRS, C-terminal EMAPII-like domain of human tyrosyl-tRNA synthetase; EELP, Entamoeba EMAPII-like polypeptide; EhKRS, lysyl-tRNA synthetase of *Entamoeba*; EhKRSΔCt, EhKRS depleted of EELP domain; HumEELP, humanized EhCtKRS (see Figure S1 and text). *** p<0.0001 vs ctl.

Human EMAPII and the EMAPII-like domain of YRS (HsCtYRS) display potent leukocyte and monocyte chemotaxis activity after proteolytic processing [22]. We evaluated whether EELP chemoattracted endothelial primary cells and monocytes in a Boyden chamber migration assay. EELP promoted migration of human vascular endothelial cells (HUVEC) as efficiently as HsEMAPII or HsCtYRS (**Figure 4A**). This effect was dependent on EELP concentration (**Figure S4**). The full length EhKRS, or its truncated form (EhKRSΔCt) did not show this effect (**Figure 4A**).

**Figure 5:**
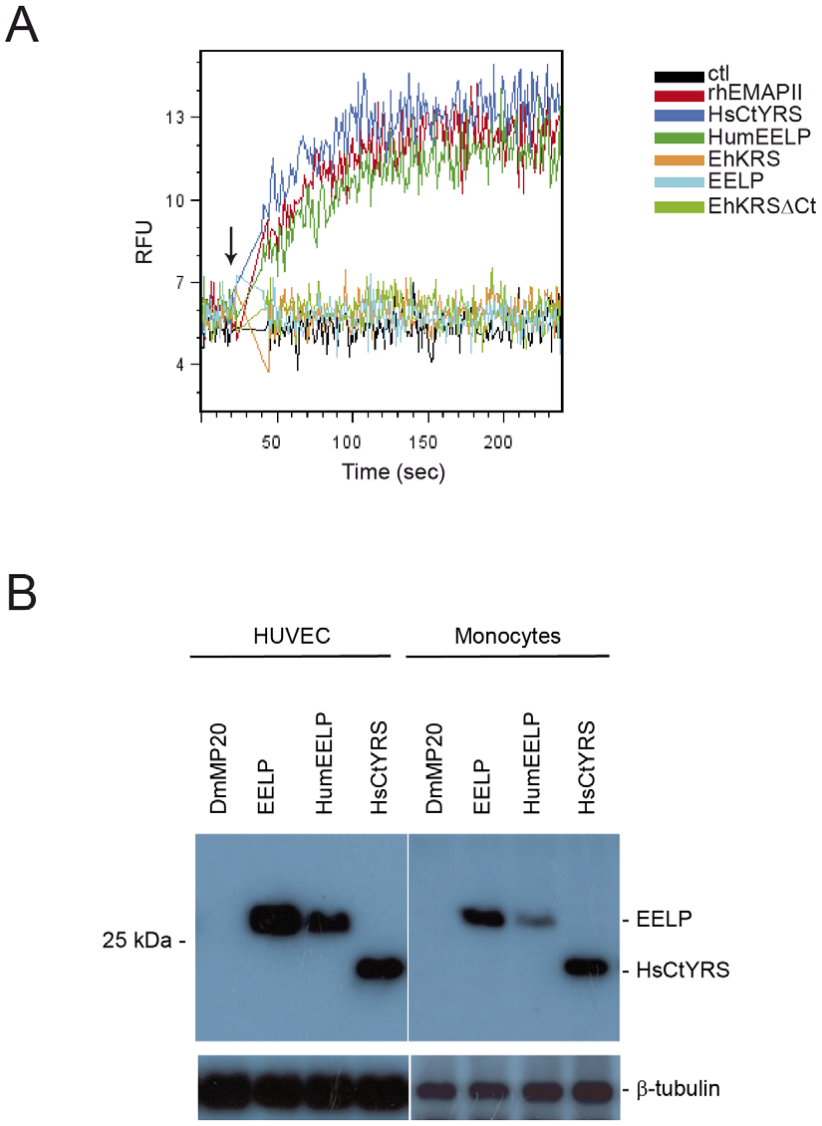
Calcium mobilisation and cellular internalization of EELP. (A) Calcium flux was observed by Fluo-4 fluorescence in monocytes stimulated with 10 nM rhEMAPII, HsCtYRS, EhKRS, EhKRSΔCt, EELP, and HumEELP. An increase in fluorescence proves increase in cytoplasmic calcium and was monitored as indicated in Methods. RFU, relative fluorescence units; the rest of abbreviations are the same as listed in Figure 4. Arrow indicates when stimuli were added. (B) EELP and HumEELP are internalized in endothelial cells and monocytes. These cells were incubated for 1 hour with 100 nM of His tagged purified proteins. Cell extracts were analyzed by western blot with α-His antibody. Control of protein loading was evaluated with α-tubulin antibody. DmMp20; recombinant *Drosophila melanogaster* Muscle protein of 25 kDa (see also Figure S1A).

We analyzed whether EELP acted in synergy with VEGF to induce HUVEC chemotaxis, but no additive activity could be seen (**Figure S5**). In contrast to HsEMAPII, EELP did not induce migration of monocytes (**Figure 4B** **and Figure S6**). Since HsEMAPII was reported to induce calcium mobilization in monocytes, we checked whether a calcium signalling was also triggered by EELP. While HsEMAPII induced calcium mobilisation in HUVEC cells (**Movie S1**) and monocytes (**Figure 5A**), EELP only mobilized calcium in endothelial cells (**Movie S2** and **Figure 5A**).

Because EELP triggers signalling in endothelial cells but not in monocytes we examined whether the chemokine binds differently to these two cell types. HUVEC and primary monocytes were incubated with purified EELP and HsCtYRS for 60 minutes, and binding was assessed by western blotting. EELP was internalized both in HUVEC and monocytes (**Figure 5B**), indicating that the different activity of EELP towards these two cell types may depend upon interactions with intracellular partners.

To understand the reason behind the functional differences between human EMAPII and EELP we compared the sequences of the two proteins. It is known that the cytokine activities of HsEMAPII and HsCtYRS depend upon a heptapeptide sequence called ‘migration motif’ [50], [51]. The only differences between this motif in human HsCtYRS and EELP are at two positions (**Figure 1B** **and Figure S1D**). Mutation of these positions in EELP to the HsCtYRS sequence turned the *Entamoeba* domain into a monocyte chemokine (**Figure 4B** **and Figure S7**) that induces calcium mobilization in these cells (**Figure 5A**). Thus, the functional differences between EELP and HsEMAPII or HsCtYRS are caused by changes in their ‘migration motifs’.

### Monocytes are chemoattracted by supernatants of EELP-stimulated HUVEC

**Figure 6:**
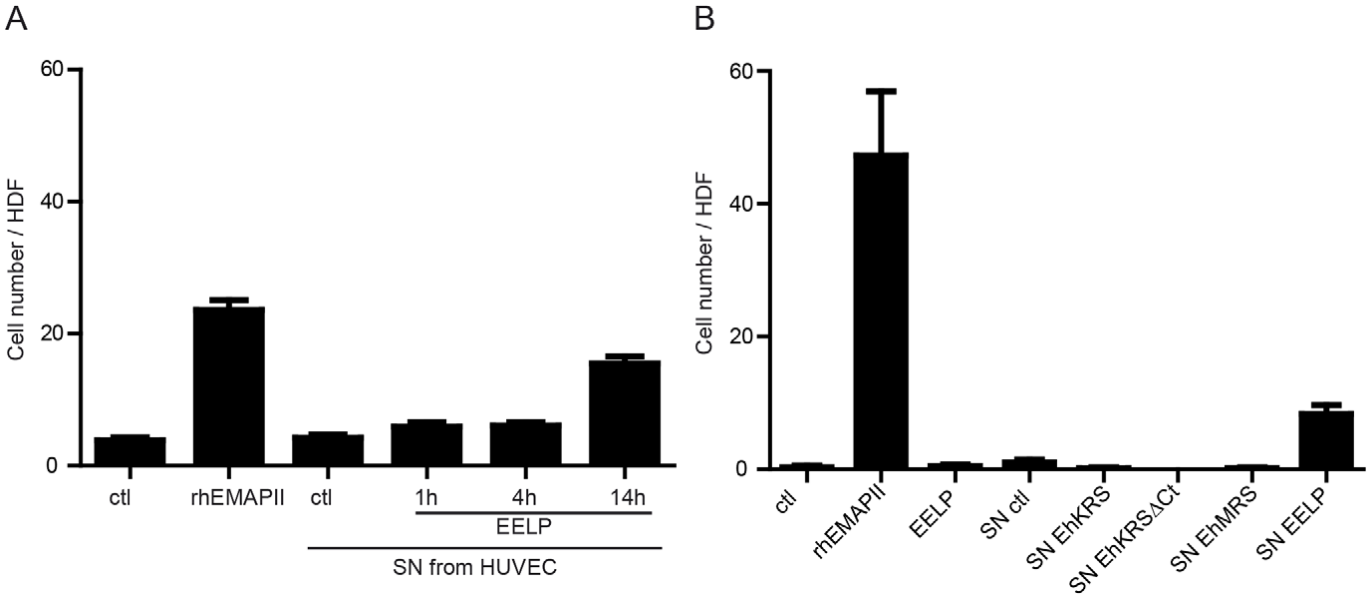
The effect of conditioned HUVEC medium on monocyte migration. (A) Number of monocytes that have migrated to 1 nM rhEMAPII or supernatants from HUVEC cultured with EELP for 1, 4 or 14 hours in the lower wells of a 24-transwell Boyden plate or (B) that have migrated to 1 nM rhEMAPII, 1 nM EELP, supernatants from HUVEC cultured for 14 hours with 1 nM EhKRS (SN EhKRS), 1 nM EhKRSΔCt (SN EhKRSΔCt) 1 nM EELP (SN EELP) or 1 nM EhMRS (SN EhMRS). Values are plotted as Mean ± SEM of three independent experiments.

We asked whether the chemoattraction of endothelial cells by EELP may represent a strategy to shield the parasite from contact with immune cells. To test this hypothesis we analyzed whether HUVEC treated with EELP generated a chemotactic response from monocytes. We incubated HUVEC with EELP for 1, 4, and 14 hours, and used the supernatants to try to induce monocyte migration. We observed that the supernatants from HUVEC cells treated with EELP for 14 hours were able to induce monocyte migration (**Figure 6A**). No chemotactic activity could be observed when we used the supernatants from HUVEC treated with EhKRS or EhKRSΔCt for 14 hours (**Figure 6B**). Thus, EELP indirectly induces monocytic migration through the action on endothelial cells.

## Discussion

New signaling domains can evolve within proteins that provide suitable structural and functional platforms for the selection of new functions. Multi domain aaRS are a clear example of a house-keeping protein family that can readily incorporate new functions through the evolution of additional domains [52]. In many cases these domains evolve from RNA-binding structures that complement the original functional core of these proteins. The emergence of cell-signaling domains within larger house-keeping enzymes may be favored by the intrinsic structural and functional stability of the later, which facilitate the development of activation mechanisms based on the action of proteases.

Multicellular organisms have evolved new signaling systems to avoid, modulate, or respond to the immune challenges built by their hosts. The nature of such strategies in the infectious cycle of *Entamoeba* has not been well explored, but the fact that the parasite is capable of traversing several tissue barriers and infect different organs argues in favor of the existence of mechanisms designed to modulate the host immune response [5]. In this context, the expression of a mimic of a human cytokine could contribute to the modulation of host signaling pathways to the parasite's advantage.

We have discovered that two aaRS from the parasite *E. histolytica* contain a C-terminal domain that structurally mimics the human cytokine EMAPII. This discovery underlines again the facility with which aaRS can evolve to incorporate signaling activities. Until now, however, aaRS-associated signaling functions had not been found to cross species barriers. Our study demonstrates that, upon exposure to inflammatory signals, *Entamoeba histolytica* overexpresses EhKRS and EhMRS. Parasite proteases and human elastase are capable of processing EhKRS and EhMRS to liberate their respective C-terminal domains, which are 99% identical in sequence and we have named as EELP. Mass spectrometry analysis demonstrates that EELP can be found in *Entamoeba* extracts, and immunolocalization studies show that EELP localizes to the peripheral area of the parasite's cytosol, in close proximity to the cell membrane.

Reported HsEMAPII effects include chemotactic attraction of monocytes, dendritic cells and endothelial cells, induction of apoptosis in lymphocytes, and TNF-α production by monocytes/macrophages [30], [53], [54]. In contrast, EELP attracts human endothelial cells but not human monocytes, and this cellular specificity depends on the sequence of the signaling peptide of this protein. In turn, endothelial cells activated by EELP generate a chemoattractant signal for monocytes.

Amebic chemoattractant activity present in non-vesicular membranes of the parasite has already been described [55], [56], and is possibly linked to the molecular interaction between the invading parasite and the immune response. In the initial interactions of *Entamoeba* with host cells in the epithelial mucosa, a local inflammatory response is mounted by the host. Activated monocytes/macrophages produce TNF-α, which induces parasite motility that is essential for pathogenic amebiasis [57]. Our data show that TNF-α up-regulates EhKRS and EhMRS gene expression, which are processed to EELP by proteases to induce chemotaxis of endothelial cells.

Activated endothelial cells produce interleukin-6 (IL-6), which, through signaling to its intracellular target STAT3, plays a central role in intestinal epithelial cell homeostasis [58], [59]. EELP could, therefore, influence the immune response through its chemotactic activity. Our current hypothesis for the biological function of EELP posits that the presence of endothelial cells attracted by the chemokine during *Entamoeba* infections directs the immune system towards a repairing response, maintaining mucosal homeostasis, preventing inflammation, and improving parasite survival and fitness.

The affinity of HsEMAPII for monocytes can be completely abolished through two residue changes in EELP without affecting the activity of either protein over endothelial cells. This fact indicates that the mechanism of action of EMAPII and EELP is complex, and coupled to cell-type specific pathways that respond differently to the sequence and/or structure of this family of cytokines.

## Supporting Information

Figure S1 **Experimental and computational comparisions of EELP and EMAPII.** (A) coomassie blue staining of purified 6His-tagged HsCtYRS, EhMRS, ,EhKRS, EELP, EhKRSΔCt, and HumEELP. DmMp20 is an unrelated protein used as reference. (B) protein sequence alignement of EhCtKRS, EhCtMRS, and human EMAPII. Both EhCtKRS and EhCtMRS are 99% identical (only Ala133 in EhCtKRS is changed to Ser in EhCtMRS) and are collectively called EELP. (C) Diagram showing the relative length of the proteins, and the position of the EMAPII domain (blue box). The red box marks the position of the heptapeptide migration motif. (D) Alignment of the heptapeptide migration motif of human EMAPII and HsCtYRS, and EELP from several *Entamoeba* species. The two residues mutated to obtain the humanized domain of EELP (HumEELP) are boxed in grey. Only the peptide migration motif is shown. For full comparison of the human and *Entamoeba* sequences see Figure 1B.
(TIF)

Figure S2 **EhKRS is upregulated by inflammation signals.** Expression of EhKRS detected by immunoblot with α-NtEhKRS antibodies. *Entamoeba* trophozoites were stimulated for 24 h with 100 ng/ml TNF-α, 100 ng/ml LPS, or co-cultured with primary monocytes (monos) or DLD1 cells pre-activated or not with 100 ng/ml LPS for 6 h. Gal/GalNAc lectin identified with the α-LGL antibody was used as a control.
(TIF)

Figure S3 **Identification of EELP in immunoprecipitated** ***Entamoeba*** **extracts by mass spectrometry.** (A) Coomassie blue stained SDS-PAGE of amoeba crude extracts immunoprecipitated with α-EELP. Arrows mark the bands corresponding to endogenous (left) and recombinant(right, 6His-tagged) EELP. (B) Peptide sequence and fragmentation spectra obtained after trypsin digestion and Nano-LC-MS/MS analysis of the band shown in Figure S3A. (C) The peptide sequences determined by mass spectrometry analysis are underlined in the EELP sequence (Coverage = 17,5 and score = 238,9.).
(TIF)

Figure S4 **Dose-dependent HUVEC migration induced by EELP.** The number of HUVEC cells that migrated in response to increasing concentrations of EELP in a Boyden chemotaxis plate assay.
(TIF)

Figure S5 **No synergistic effect of EELP and VEGF over HUVEC migration.** The number of HUVEC cells that migrated in response to increasing concentrations of VEGF in the presence (1 nM), or absence of EELP, in a Boyden chemotaxis plate assay.
(TIF)

Figure S6 **Lack of EELP chemotaxis activity towards monocytes.** The number of primary human monocytes that migrated in response to 1 nM rhEMAPII, 1 nM HsCtYRS, or increasing concentrations of EELP, in a Boyden chemotaxis plate assay.
(TIF)

Figure S7 **Humanized EELP recovers chemotaxis activity towards monocytes.** The number of human primary monocytes that migrated in response to EELP, humanized EELP (humEELP), rhEMAPII and HsCtYRS. The top panels show representative microphotographs for each condition. The bottom panel shows a histogram from 3 independent experiments. Data are plotted as mean ± SEM.
(TIF)

Movie S1 **Calcium influx by rhEMAPII.** Fluo-4 stained HUVEC cells recorded with a confocal microscope spinning disk and stimulated with 10 nM rhEMAPII. Time is accelerated 10 fold. Scale bar, 7 µm.
(AVI)

Movie S2 **Calcium influx by EELP.** Fluo-4 stained HUVEC cells recorded with a confocal microscope spinning disk and stimulated with 10 nM EELP. Time is accelerated 10 fold. Scale bar, 7 µm.
(AVI)

Methods S1 Sequence of primers used in this study.
(DOC)
