# Correction: *Entamoeba* lysyl-tRNA Synthetase Contains a Cytokine-Like Domain with Chemokine Activity towards Human Endothelial Cells

**DOI:** 10.1371/journal.pntd.0012047

**Published:** 2024-03-19

**Authors:** Manuel Castro de Moura, Francesc Miro, Jung Min Han, Sunghoon Kim, Antonio Celada, Lluís Ribas de Pouplana

In Fig 4B of this article [1] the rhEMAPII image was erroneously duplicated in the HsCtYRS panel. A corrected version of Fig 4 is provided here, and S1 File includes images of both conditions from two different experiments. Both rhEMAPII and HsCtYRS are considered positive controls used to show the different behavior between the human EMAPII and EELP domains.

**Fig 4:**
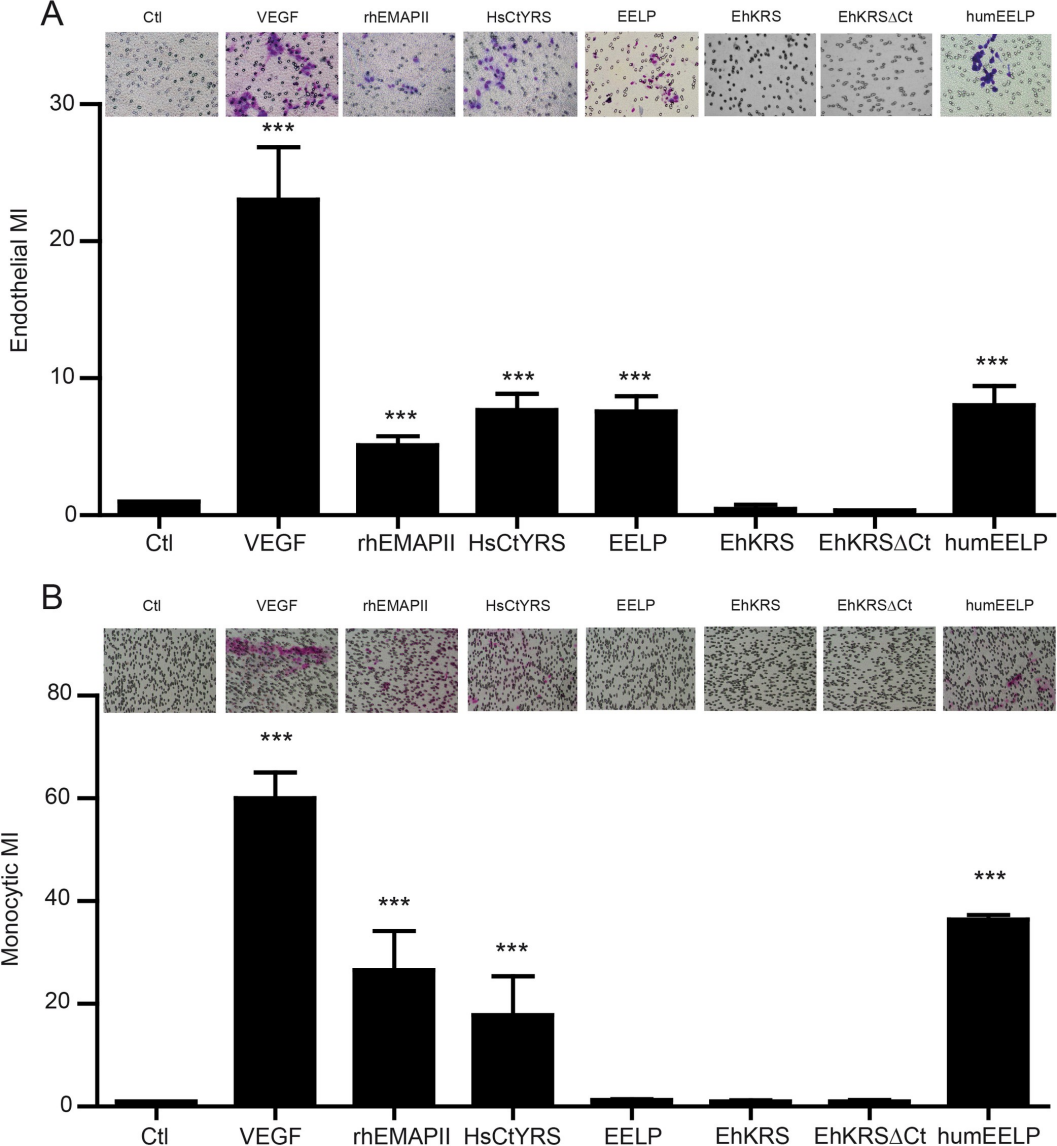
Effect of EELP on host cell migration. (A) Endothelial cell and (B) monocyte chemotaxis towards different EMAPII bearing proteins. VEGF (1 nM) was used as a common chemoattractant for both cells. Data are the mean ± SEM for at least three different experiments. Inserts show a representative photograph of cell migration. Pore membranes for HUVEC and monocyte migration were of 8 μm diameter and 5 μm diameter, respectively. Cells are stained in purple. Migration is plotted as Migration Index (MI; number of cells migrating in each condition/number of cells migrating in basal medium). Ctl, basal medium in the lower well; VEGF, vascular endothelial grothw factor; rhEMAPII, recombinant human EMAPII; HsCtYRS, C-terminal EMAPII-like domain of human tyrosyl-tRNA synthetase; EELP, *Entamoeba* EMAPII-like polypeptide; EhKRS, lysyl-tRNA synthetase of Entamoeba; EhKRSΔCt, EhKRS depleted of EELP domain; HumEELP, humanized EhCtKRS (see Fig S1 and text). *** p<0.0001 vs ctl.

## Supporting information

S1 File Representative image data from two replicates of the Fig 4 experiment. (ZIP)
