# Supplementary figures and images for: Entamoeba lysyl-tRNA Synthetase Contains a Cytokine-Like Domain with Chemokine Activity towards Human Endothelial Cells
Source: PLoS Negl Trop Dis. 2011 Nov 29;5(11):e1398. doi: 10.1371/journal.pntd.0001398 (PMC3226552; doi:10.1371/journal.pntd.0001398)

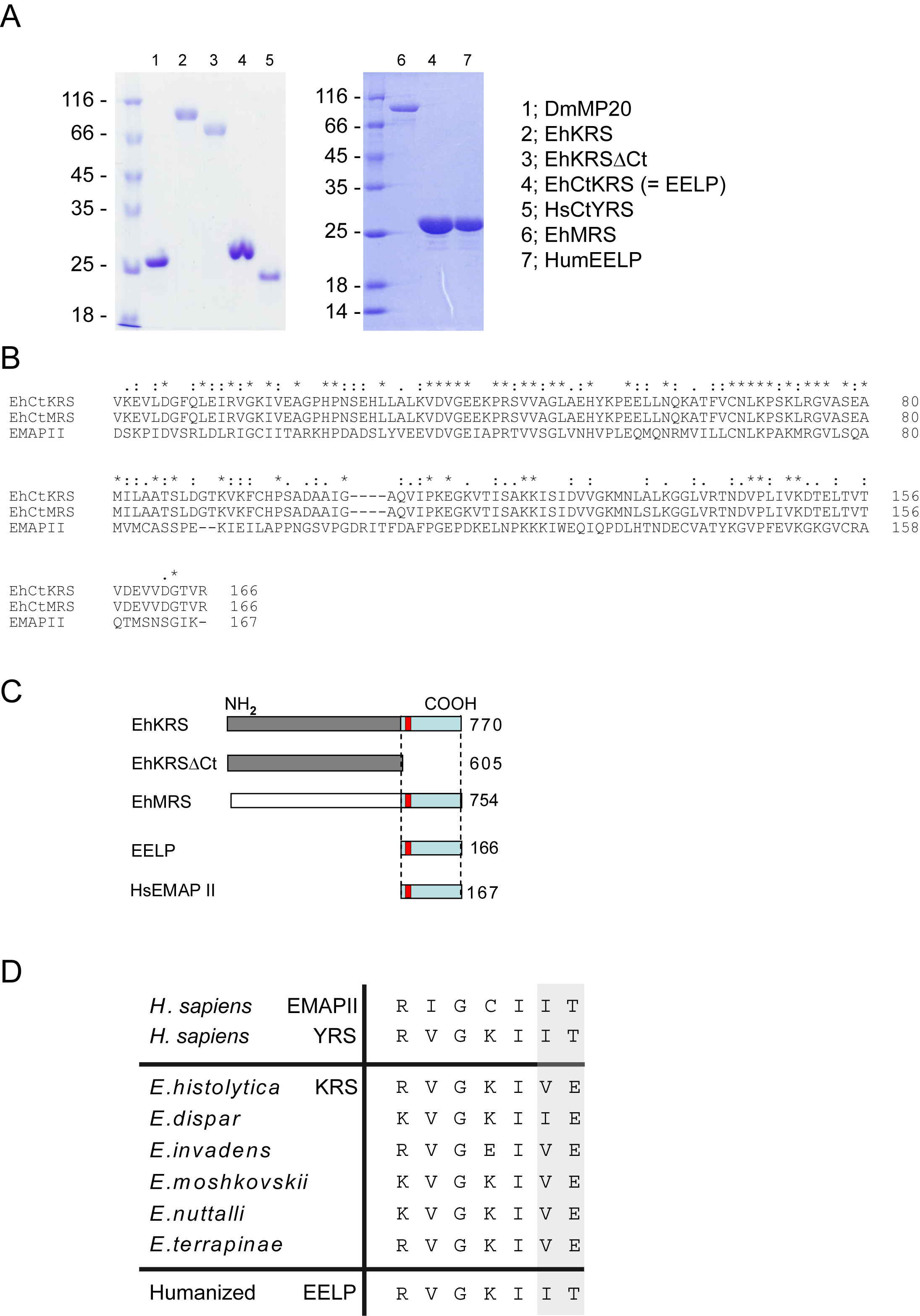

Supplement: Figure S1 — Experimental and computational comparisions of EELP and EMAPII. (A) coomassie blue staining of purified 6His-tagged HsCtYRS, EhMRS, ,EhKRS, EELP, EhKRSΔCt, and HumEELP. DmMp20 is an unrelated protein used as reference. (B) protein sequence alignement of EhCtKRS, EhCtMRS, and human EMAPII. Both EhCtKRS and EhCtMRS are 99% identical (only Ala133 in EhCtKRS is changed to Ser in EhCtMRS) and are collectively called EELP. (C) Diagram showing the relative length of the proteins, and the position of the EMAPII domain (blue box). The red box marks the position of the heptapeptide migration motif. (D) Alignment of the heptapeptide migration motif of human EMAPII and HsCtYRS, and EELP from several Entamoeba species. The two residues mutated to obtain the humanized domain of EELP (HumEELP) are boxed in grey. Only the peptide migration motif is shown. For full comparison of the human and Entamoeba sequences see Figure 1B. (TIF) [file pntd.0001398.s001.tif]

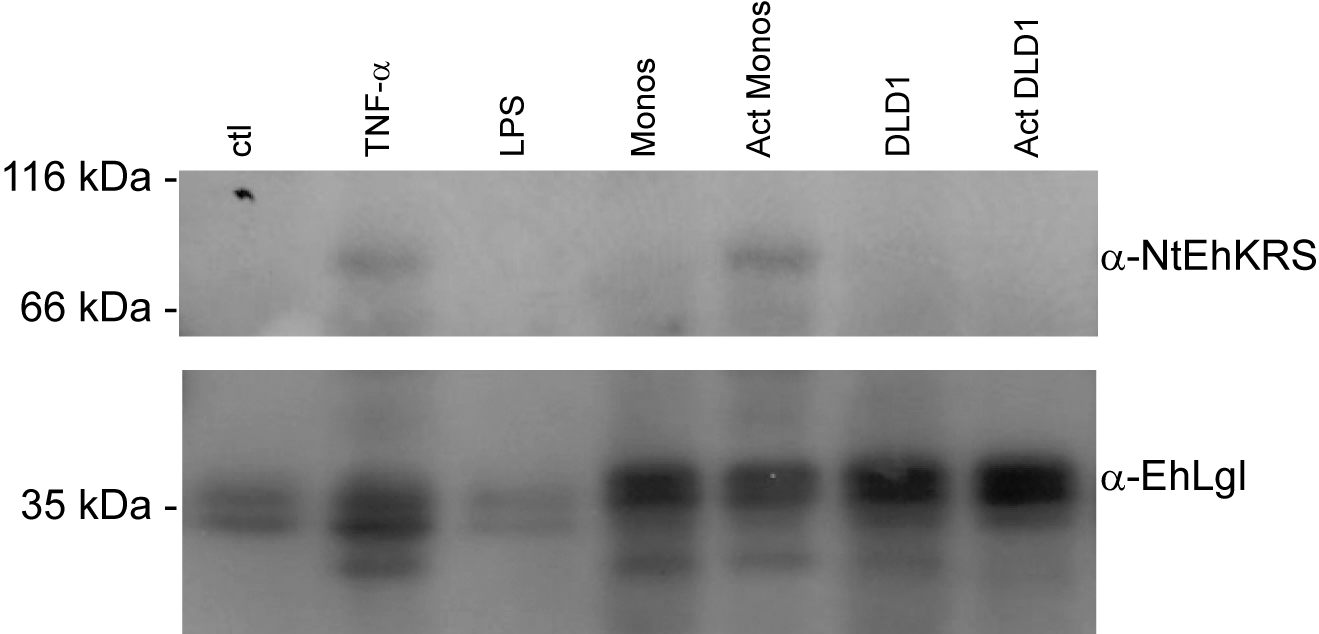

Supplement: Figure S2 — EhKRS is upregulated by inflammation signals. Expression of EhKRS detected by immunoblot with α-NtEhKRS antibodies. Entamoeba trophozoites were stimulated for 24 h with 100 ng/ml TNF-α, 100 ng/ml LPS, or co-cultured with primary monocytes (monos) or DLD1 cells pre-activated or not with 100 ng/ml LPS for 6 h. Gal/GalNAc lectin identified with the α-LGL antibody was used as a control. (TIF) [file pntd.0001398.s002.tif]

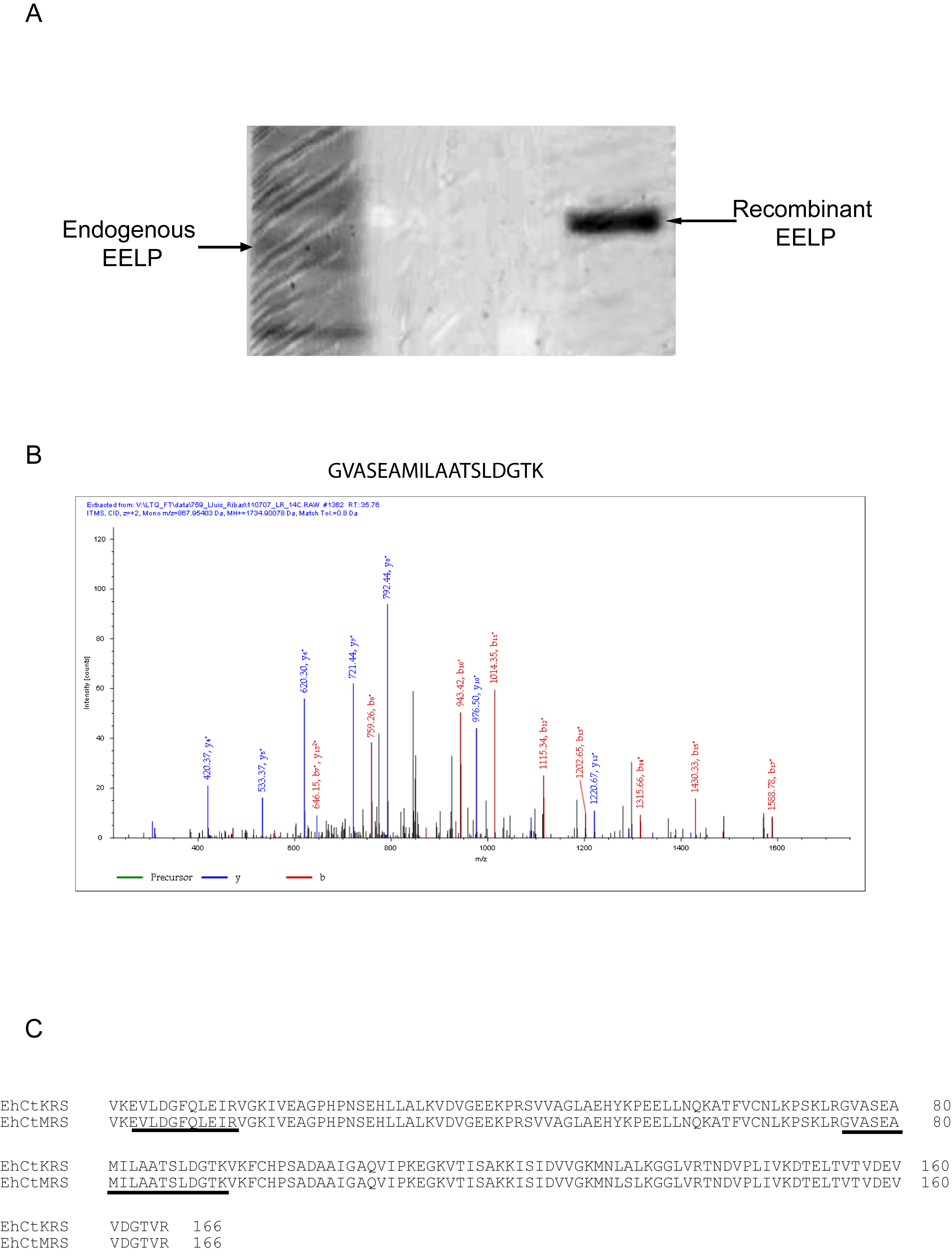

Supplement: Figure S3 — Identification of EELP in immunoprecipitated Entamoeba extracts by mass spectrometry. (A) Coomassie blue stained SDS-PAGE of amoeba crude extracts immunoprecipitated with α-EELP. Arrows mark the bands corresponding to endogenous (left) and recombinant(right, 6His-tagged) EELP. (B) Peptide sequence and fragmentation spectra obtained after trypsin digestion and Nano-LC-MS/MS analysis of the band shown in Figure S3A. (C) The peptide sequences determined by mass spectrometry analysis are underlined in the EELP sequence (Coverage = 17,5 and score = 238,9.). (TIF) [file pntd.0001398.s003.tif]

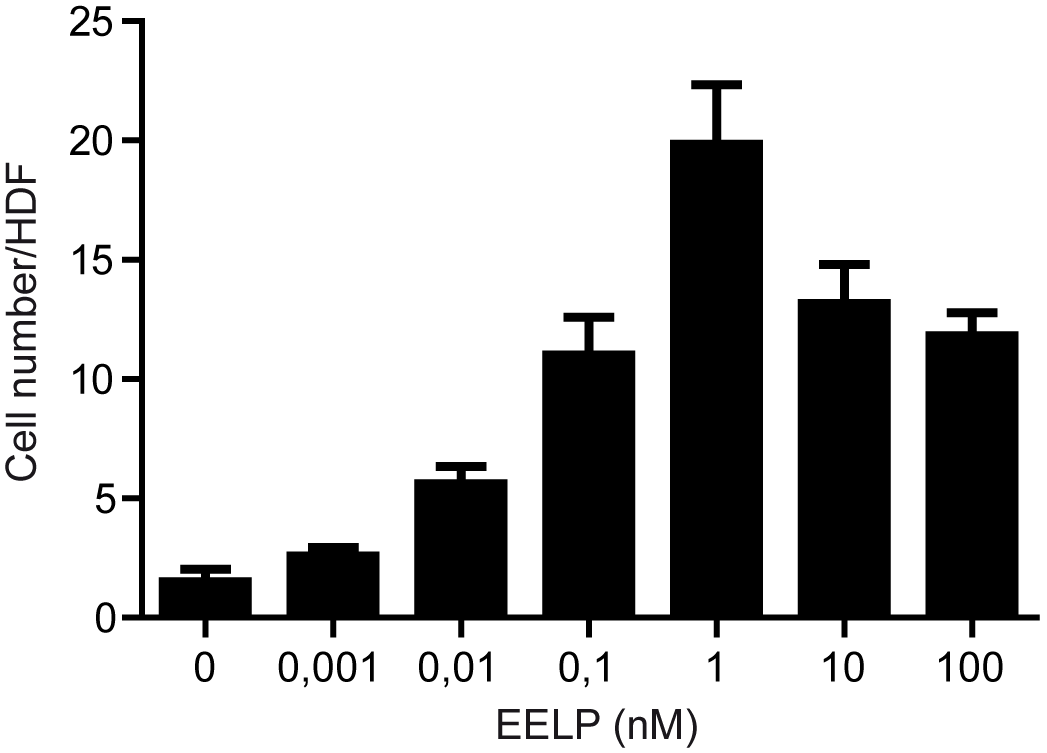

Supplement: Figure S4 — Dose-dependent HUVEC migration induced by EELP. The number of HUVEC cells that migrated in response to increasing concentrations of EELP in a Boyden chemotaxis plate assay. (TIF) [file pntd.0001398.s004.tif]

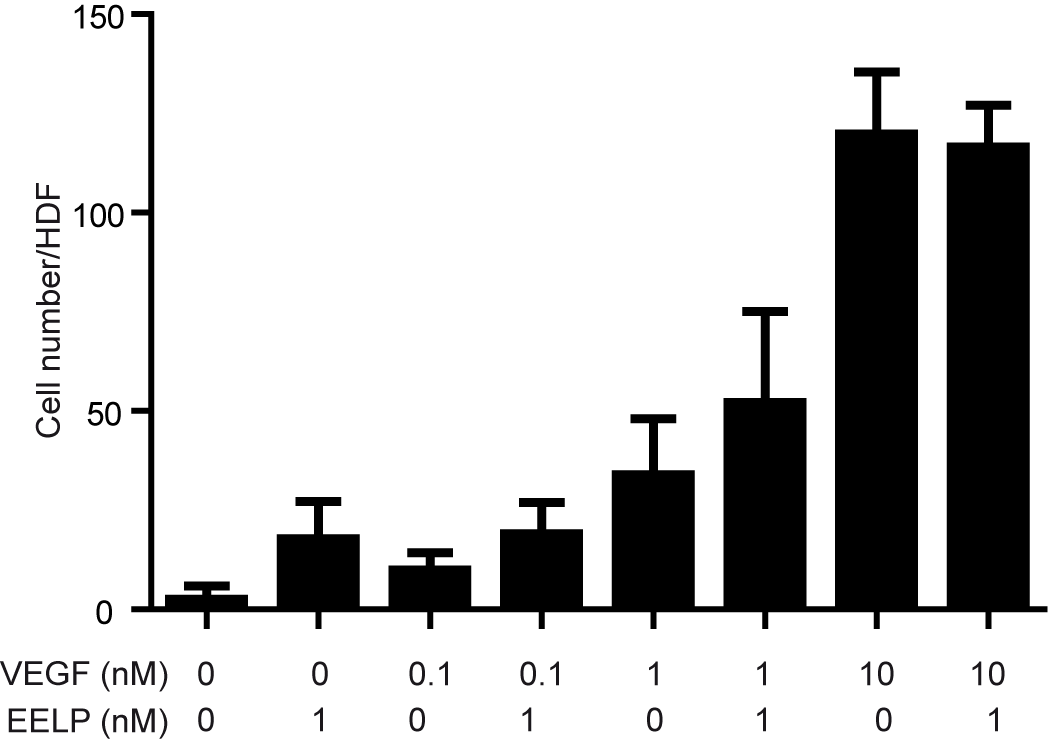

Supplement: Figure S5 — No synergistic effect of EELP and VEGF over HUVEC migration. The number of HUVEC cells that migrated in response to increasing concentrations of VEGF in the presence (1 nM), or absence of EELP, in a Boyden chemotaxis plate assay. (TIF) [file pntd.0001398.s005.tif]

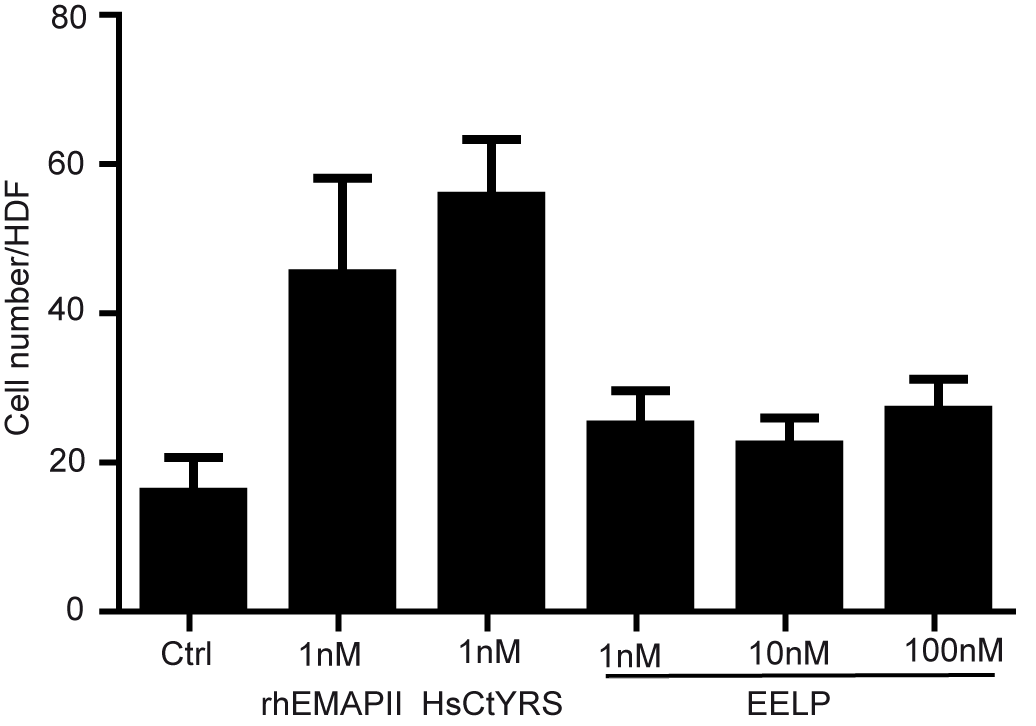

Supplement: Figure S6 — Lack of EELP chemotaxis activity towards monocytes. The number of primary human monocytes that migrated in response to 1 nM rhEMAPII, 1 nM HsCtYRS, or increasing concentrations of EELP, in a Boyden chemotaxis plate assay. (TIF) [file pntd.0001398.s006.tif]

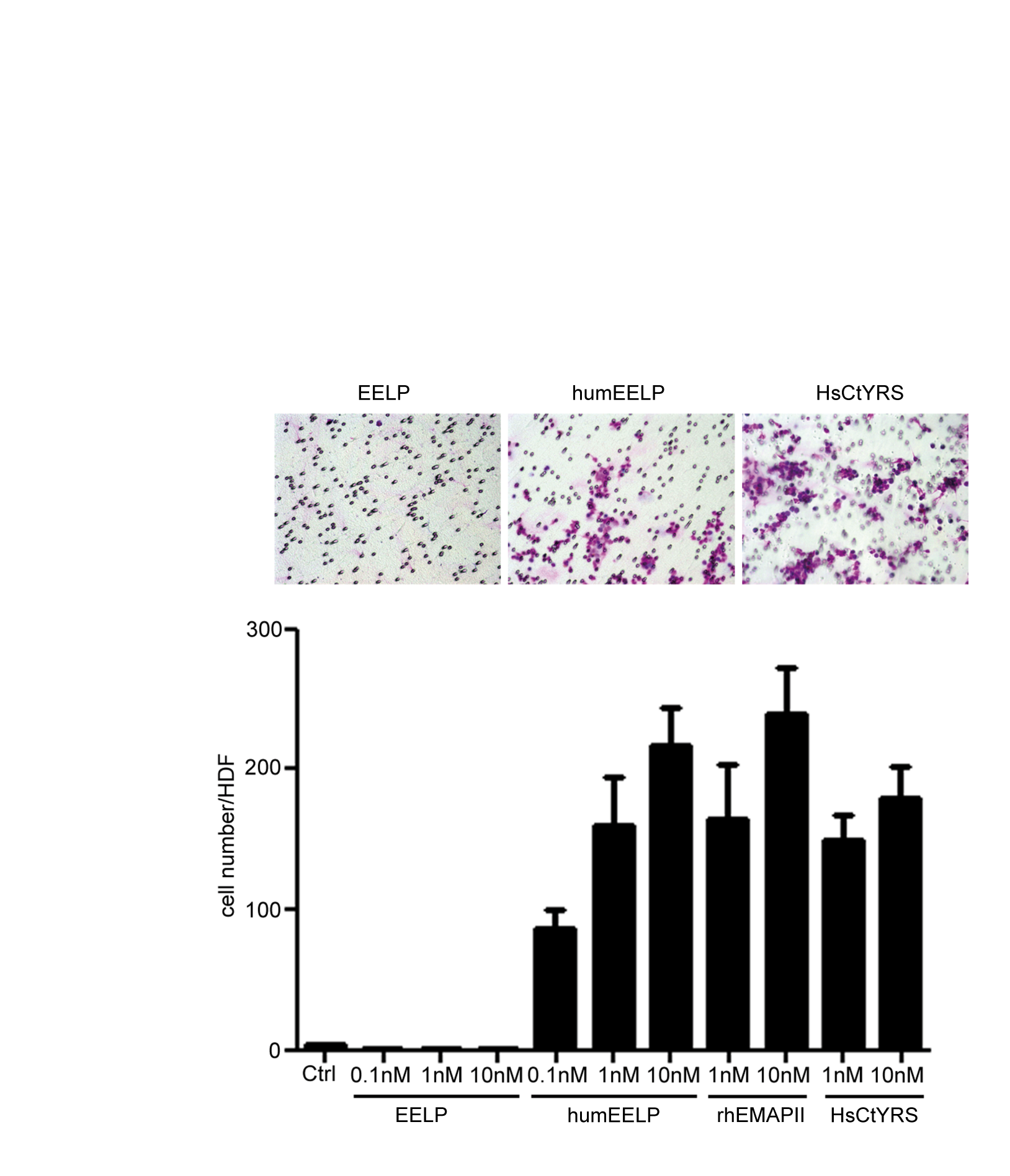

Supplement: Figure S7 — Humanized EELP recovers chemotaxis activity towards monocytes. The number of human primary monocytes that migrated in response to EELP, humanized EELP (humEELP), rhEMAPII and HsCtYRS. The top panels show representative microphotographs for each condition. The bottom panel shows a histogram from 3 independent experiments. Data are plotted as mean ± SEM. (TIF) [file pntd.0001398.s007.tif]
