# Supplementary material for: Entamoeba lysyl-tRNA Synthetase Contains a Cytokine-Like Domain with Chemokine Activity towards Human Endothelial Cells
Source: PLoS Negl Trop Dis. 2011 Nov 29;5(11):e1398. doi: 10.1371/journal.pntd.0001398 (PMC3226552; doi:10.1371/journal.pntd.0001398)
Supplement: Methods S1 — Sequence of primers used in this study. (DOC) [file pntd.0001398.s010.doc]

**SUPPORTING INFORMATION**

**METHODS S1**

Sequence of primers used for DNA amplification of *KRS* and *MRS* of Entamoeba species:

KRSdegF, 5'-ATGKCWAARMAACTCTTCCTC;

KRSdegR, 5'-TTATCTRACKGTTCCATCAAYAA;

MRSF, 5'-ATGGCTACTCAAGTTAAACCATC;

MRSdegR, 5'-TYATCTRACRGTTCCATCAACWAC

Sequence of primers used for cloning *E. histolytica KRS* gene:

EhKRSPETF,

5'-GACGACGACAAGATGTCTAAACAACTCTTCCTCAATAGA;

EhKRSPETR,

5'-GAGGAGAAGCCCGGTTTATCTAACGGTTCCATCAACAACTTCATC.

Sequence of primers used for qRT-PCR:

EhKRSF, 5’- GAGGATGGGGACTTGGAATAG;

EhKRSR 5’- CTGGTCTCATAGTTGGGAATAAAA;

EhMRSF 5’-GTAATGCCCCTGGTGCTCGTG;

EhMRSR 5’-TGGCCATTCATATTTACTCATTGTTTCAT;

EhTRSF 5’-ATTTGGACGGAACTGCTACTGGAGA;

EhTRSR 5’-CTGGCGAAAGAACATGGAAGAAGAAAAC;
