# Supplementary figures and images for: Correction: Entamoeba lysyl-tRNA Synthetase Contains a Cytokine-Like Domain with Chemokine Activity towards Human Endothelial Cells
Source: PLoS Negl Trop Dis. 2024 Mar 19;18(3):e0012047. doi: 10.1371/journal.pntd.0012047 (PMC10950210; doi:10.1371/journal.pntd.0012047)

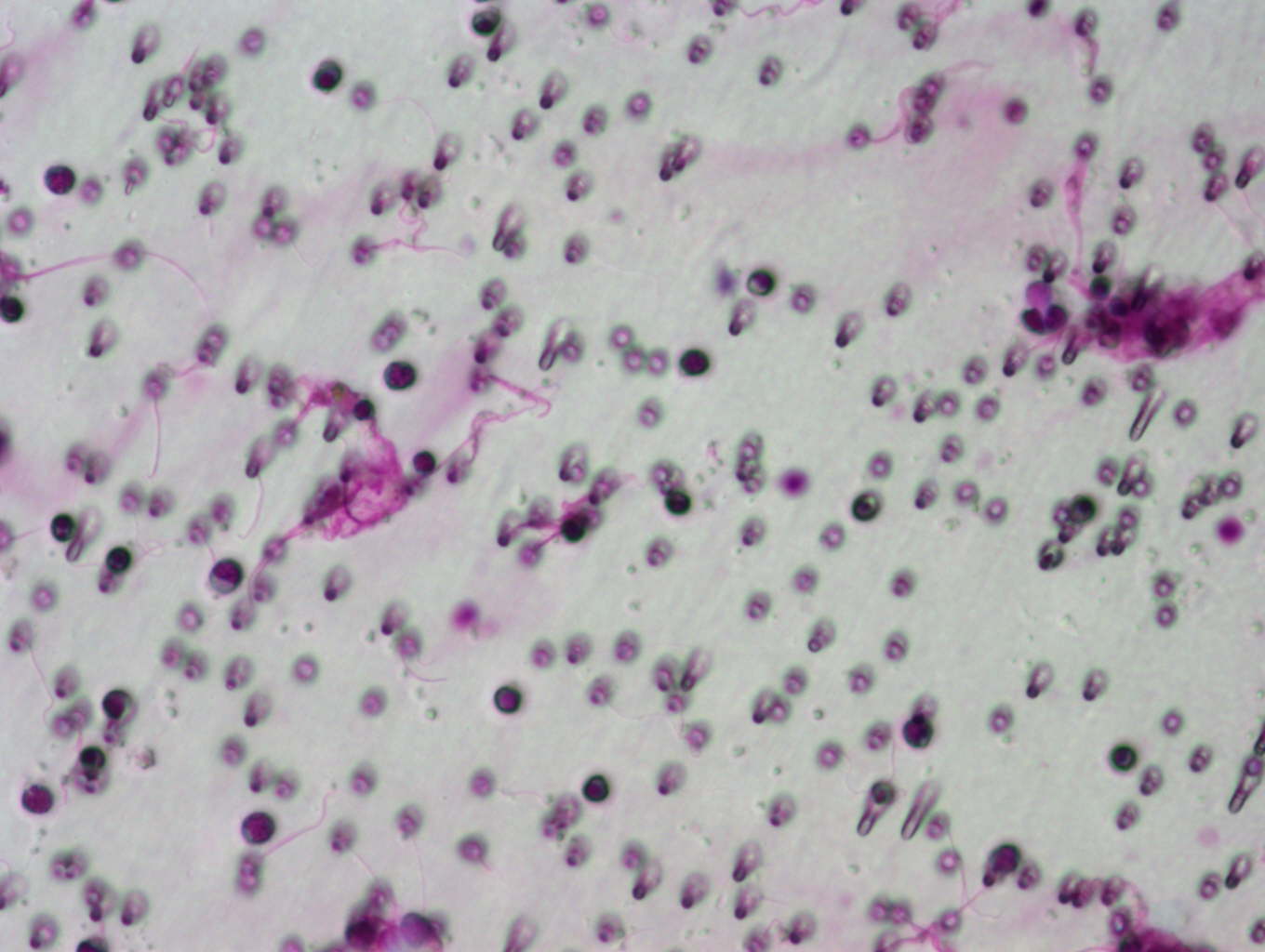

Supplement: S1 File — (ZIP) [file pntd.0012047.s001.zip › CtYRS_1.jpg]

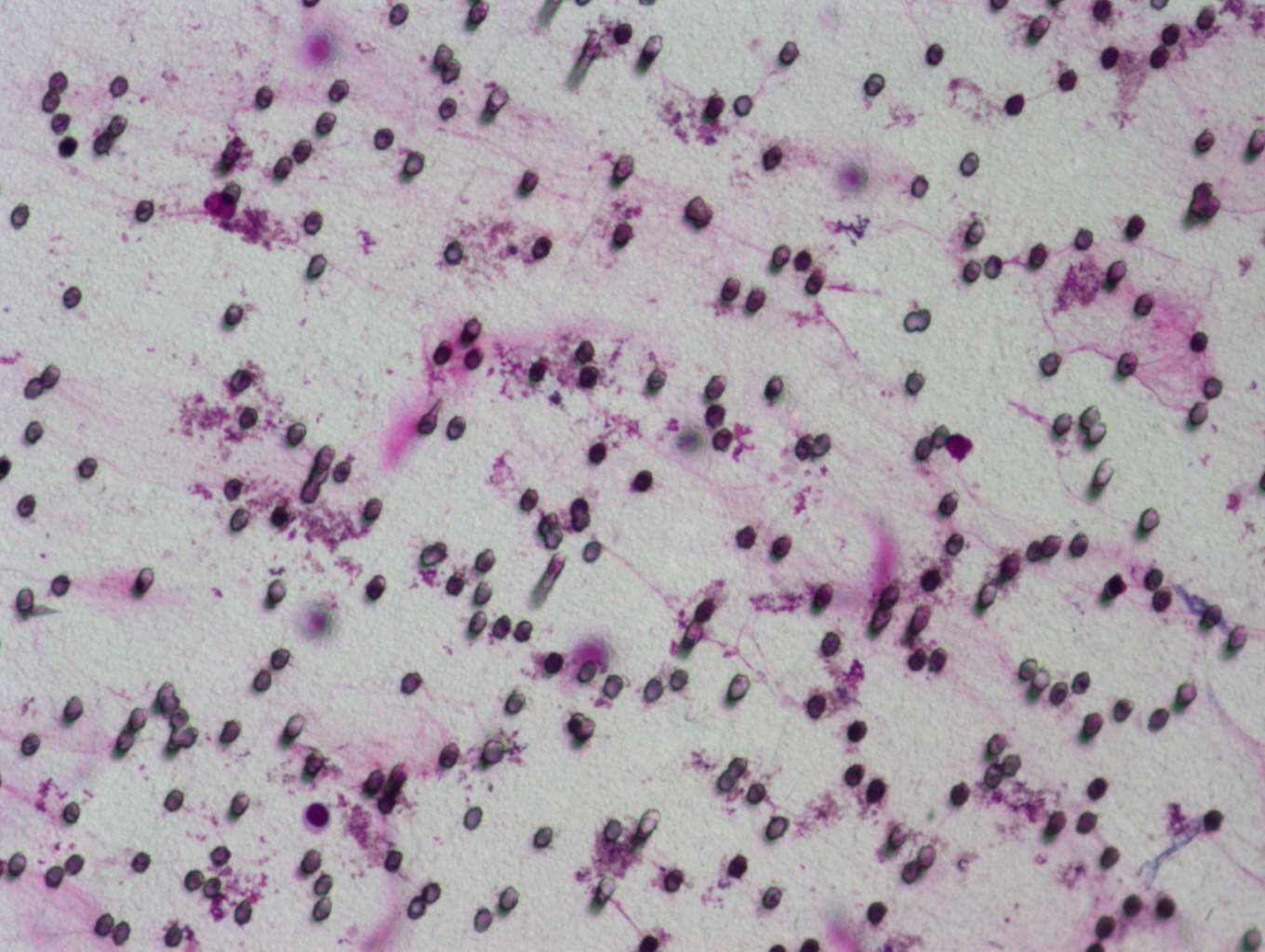

Supplement: S1 File — (ZIP) [file pntd.0012047.s001.zip › CtYRS_2.jpg]

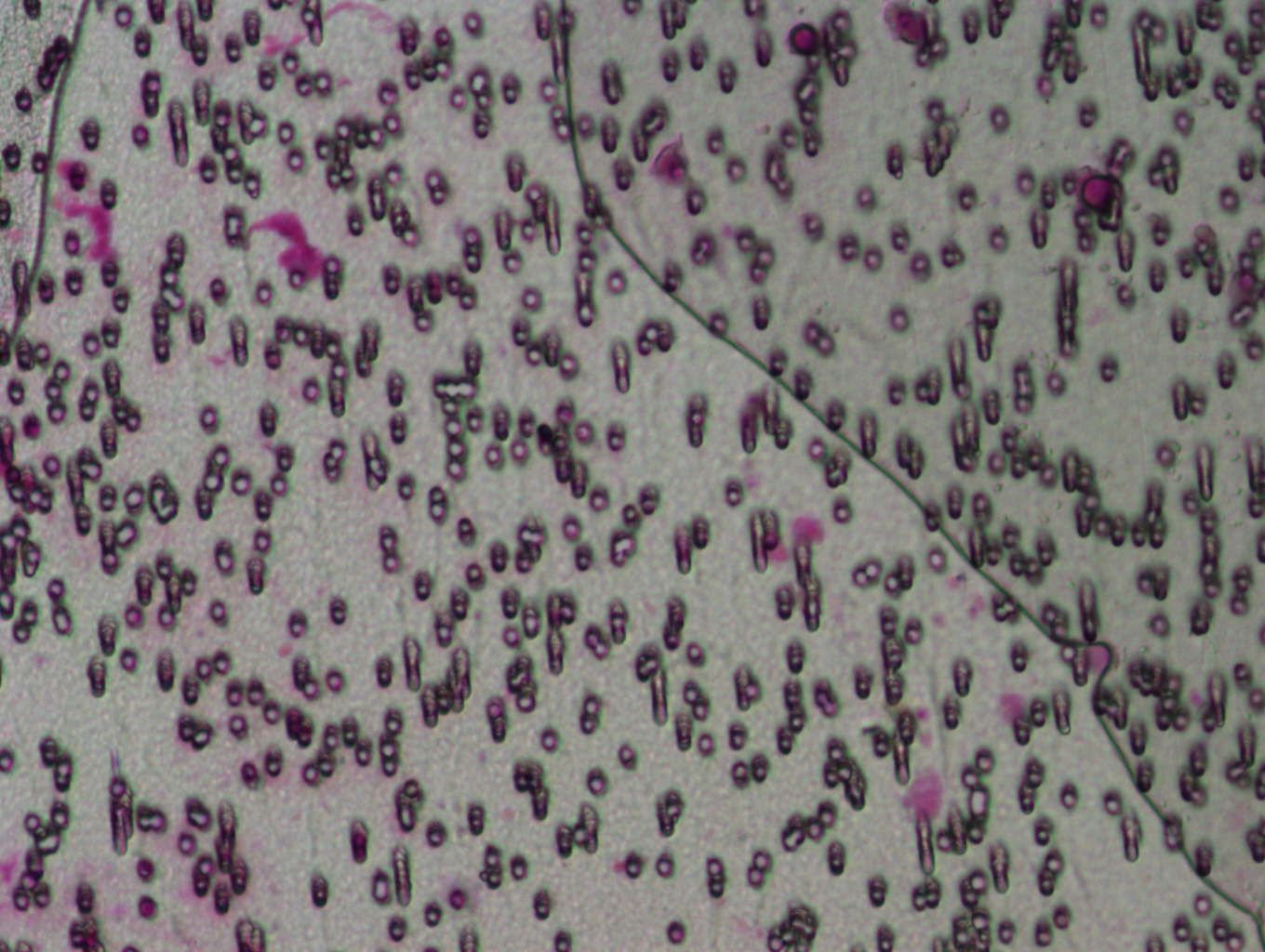

Supplement: S1 File — (ZIP) [file pntd.0012047.s001.zip › CtYRS_3.jpg]

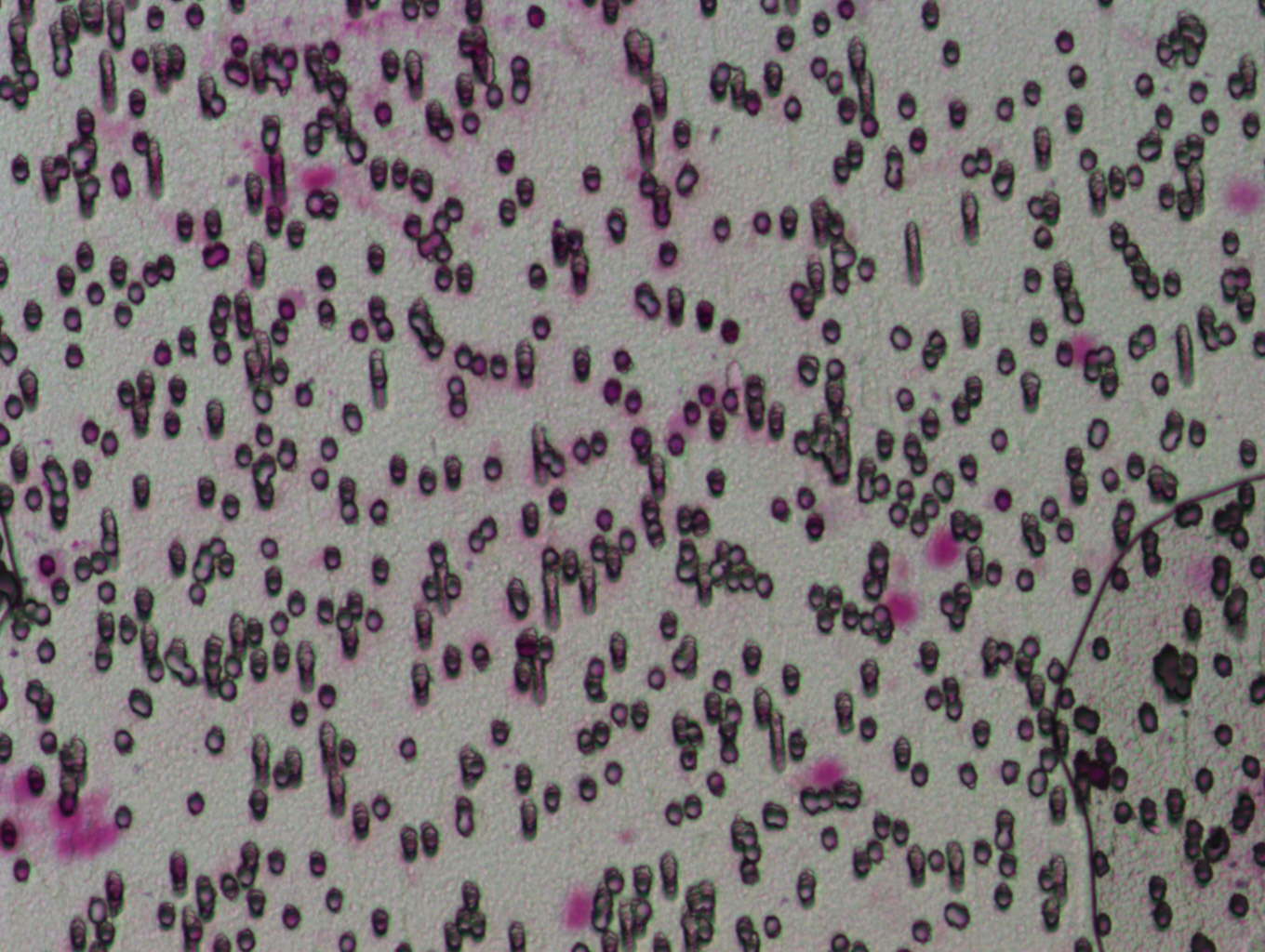

Supplement: S1 File — (ZIP) [file pntd.0012047.s001.zip › CtYRS_4.jpg]

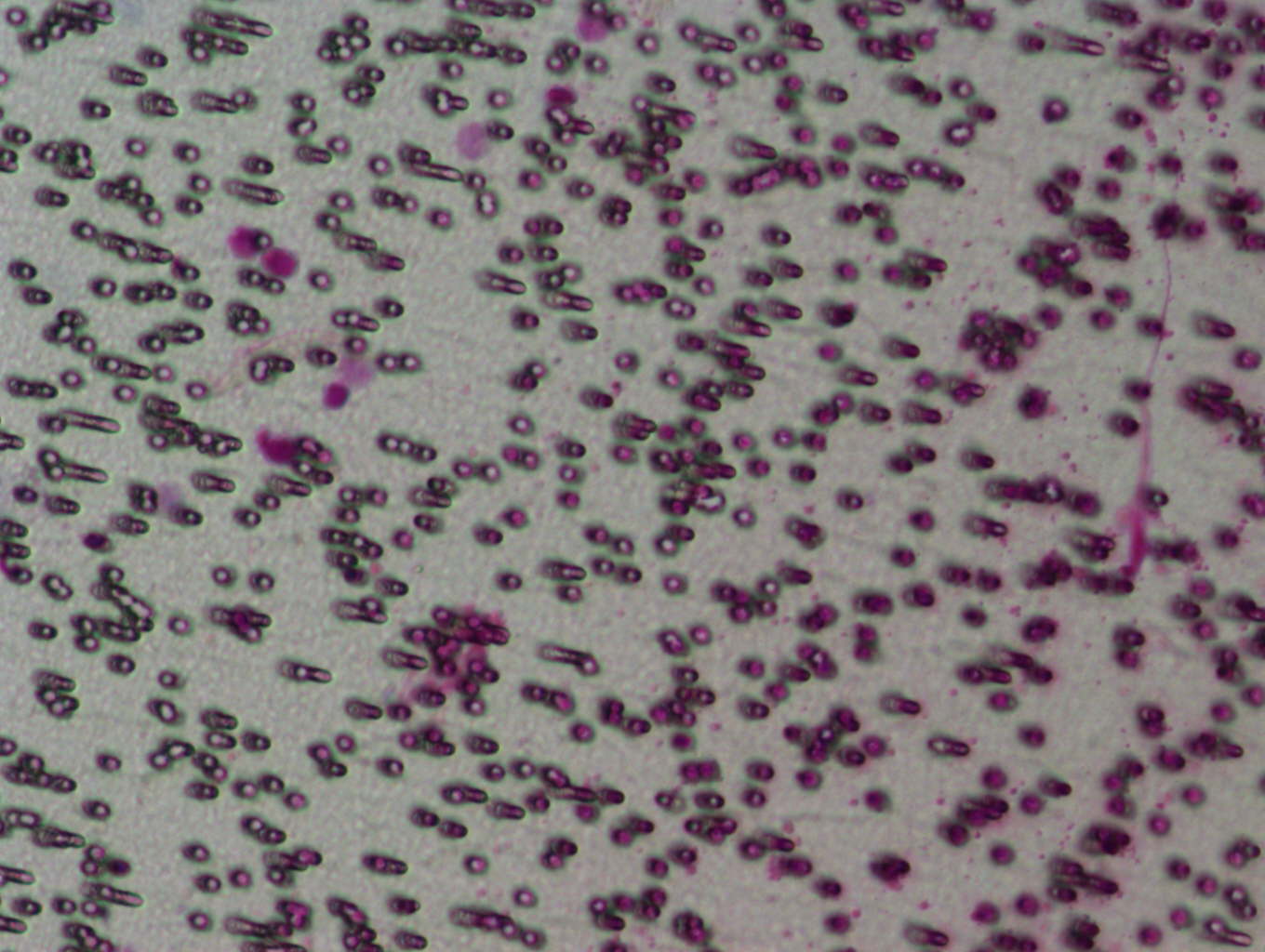

Supplement: S1 File — (ZIP) [file pntd.0012047.s001.zip › HsEMAPII_3.jpg]

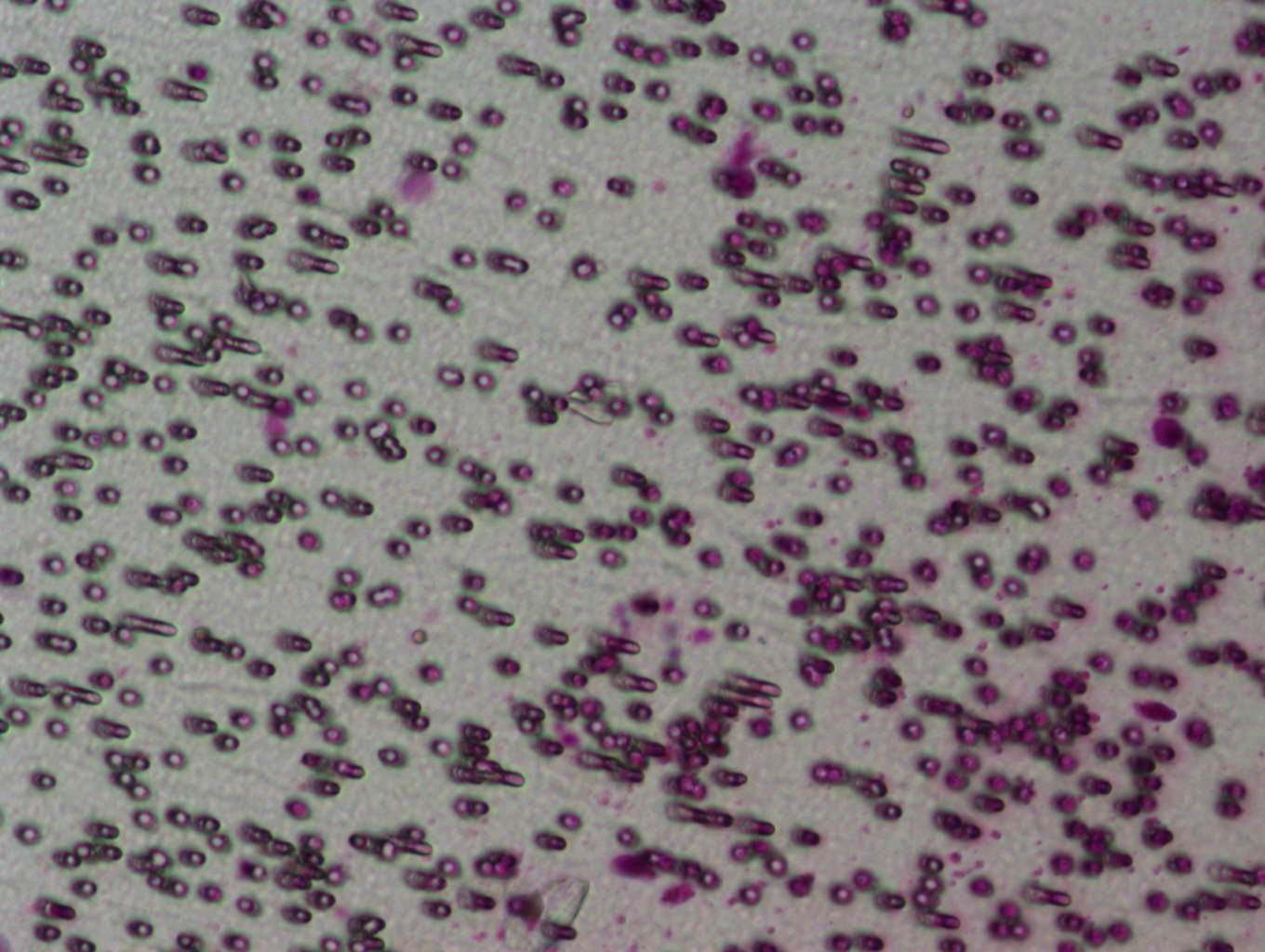

Supplement: S1 File — (ZIP) [file pntd.0012047.s001.zip › HsEMAPII_4.jpg]

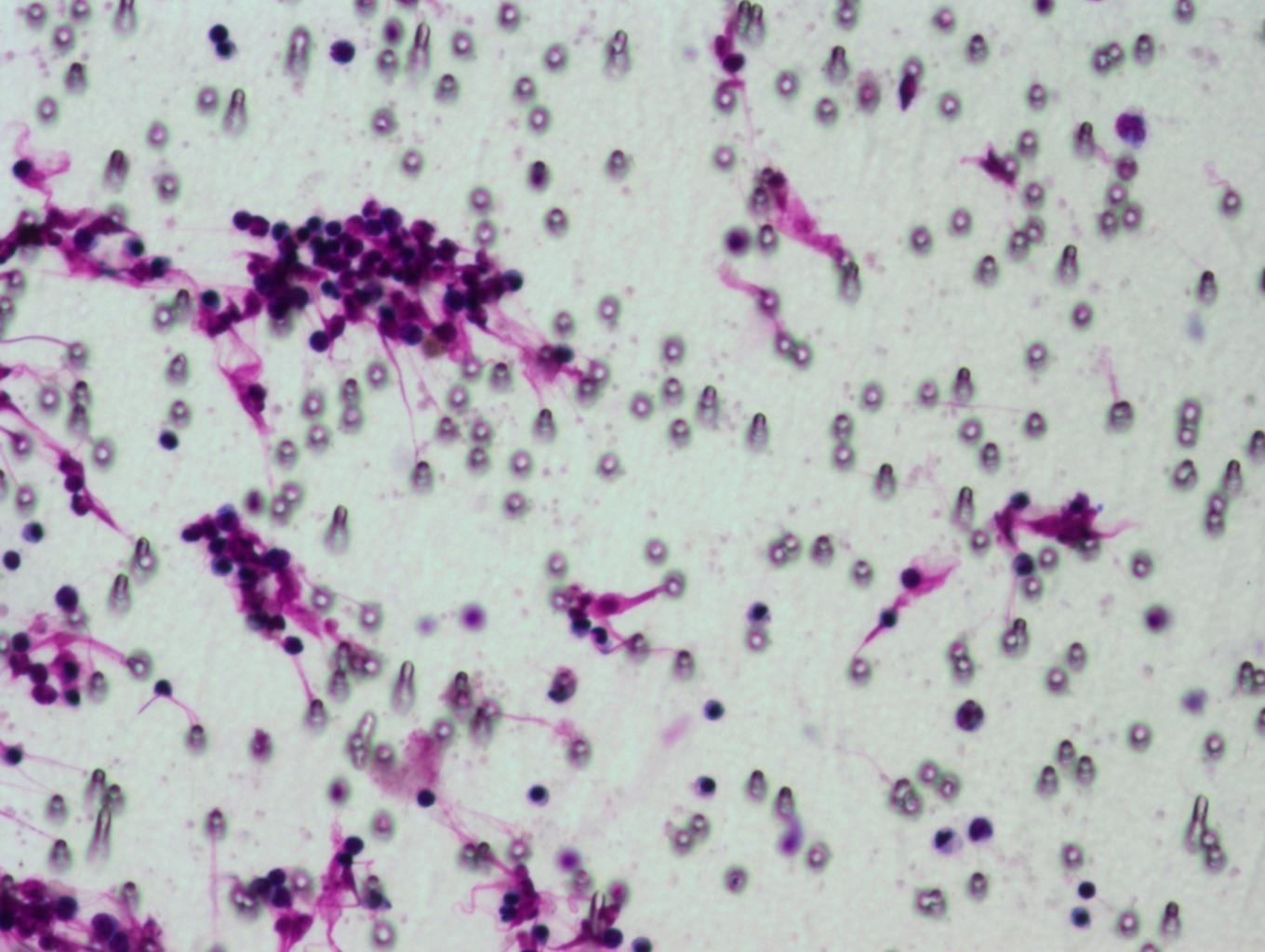

Supplement: S1 File — (ZIP) [file pntd.0012047.s001.zip › HsEMAP_1.tif]

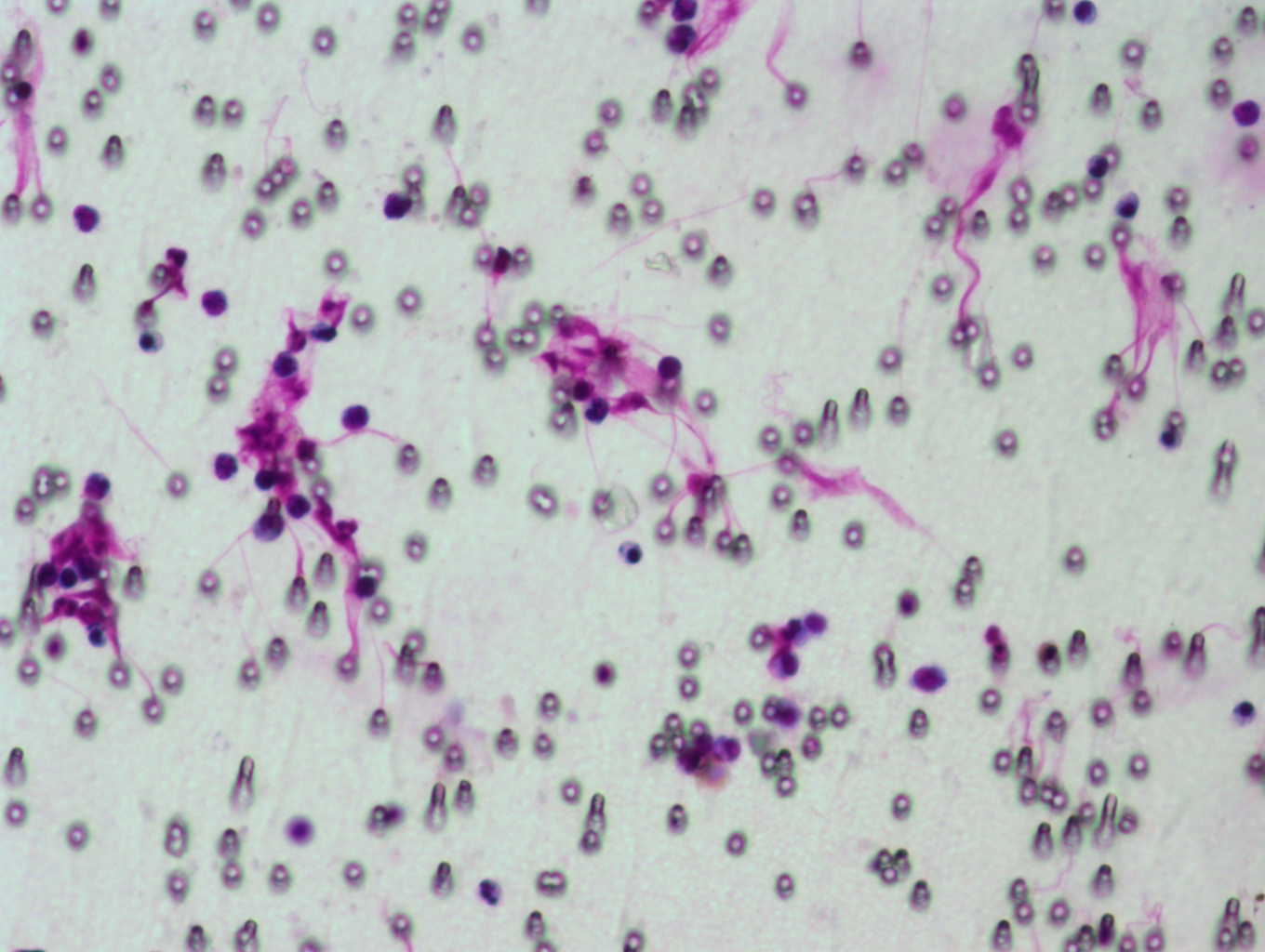

Supplement: S1 File — (ZIP) [file pntd.0012047.s001.zip › HsEMAP_2.tif]
